# Proteomic Analyses Reveal High Expression of Decorin and Endoplasmin (HSP90B1) Are Associated with Breast Cancer Metastasis and Decreased Survival

**DOI:** 10.1371/journal.pone.0030992

**Published:** 2012-02-20

**Authors:** Thomas R. Cawthorn, Juan C. Moreno, Moyez Dharsee, Danh Tran-Thanh, Suzanne Ackloo, Pei Hong Zhu, Girish Sardana, Jian Chen, Peter Kupchak, Lindsay M. Jacks, Naomi A. Miller, Bruce J. Youngson, Vladimir Iakovlev, Cynthia J. Guidos, Katherine A. Vallis, Kenneth R. Evans, David McCready, Wey L. Leong, Susan J. Done

**Affiliations:** 1 Campbell Family Institute for Breast Cancer Research, University Health Network, Toronto, Ontario, Canada; 2 Laboratory Medicine Program, University Health Network, Toronto, Ontario, Canada; 3 Ontario Cancer Biomarker Network, Toronto, Ontario, Canada; 4 Department of Biostatistics, University Health Network, Toronto, Ontario, Canada; 5 Department of Medical Biophysics, University of Toronto, Toronto, Ontario, Canada; 6 Department of Laboratory Medicine and Pathobiology, University of Toronto, Toronto, Ontario, Canada; 7 Department of Laboratory Medicine St. Michael's Hospital, Toronto, Ontario, Canada; 8 Program in Developmental and Stem Cell Biology, Hospital for Sick Children Research Institute, and Department of Immunology, University of Toronto, Toronto, Ontario, Canada; 9 MRC/CRUK Gray Institute for Radiation Oncology and Biology, University of Oxford, Oxford, United Kingdom; 10 Department of Surgical Oncology, University Health Network, Toronto, Ontario, Canada; Health Canada, Canada

## Abstract

**Background:**

Breast cancer is the most common malignancy among women worldwide in terms of incidence and mortality. About 10% of North American women will be diagnosed with breast cancer during their lifetime and 20% of those will die of the disease. Breast cancer is a heterogeneous disease and biomarkers able to correctly classify patients into prognostic groups are needed to better tailor treatment options and improve outcomes. One powerful method used for biomarker discovery is sample screening with mass spectrometry, as it allows direct comparison of protein expression between normal and pathological states. The purpose of this study was to use a systematic and objective method to identify biomarkers with possible prognostic value in breast cancer patients, particularly in identifying cases most likely to have lymph node metastasis and to validate their prognostic ability using breast cancer tissue microarrays.

**Methods and Findings:**

Differential proteomic analyses were employed to identify candidate biomarkers in primary breast cancer patients. These analyses identified decorin (DCN) and endoplasmin (HSP90B1) which play important roles regulating the tumour microenvironment and in pathways related to tumorigenesis. This study indicates that high expression of Decorin is associated with lymph node metastasis (p<0.001), higher number of positive lymph nodes (p<0.0001) and worse overall survival (p = 0.01). High expression of HSP90B1 is associated with distant metastasis (p<0.0001) and decreased overall survival (p<0.0001) these patients also appear to benefit significantly from hormonal treatment.

**Conclusions:**

Using quantitative proteomic profiling of primary breast cancers, two new promising prognostic and predictive markers were found to identify patients with worse survival. In addition HSP90B1 appears to identify a group of patients with distant metastasis with otherwise good prognostic features.

## Introduction

Breast cancer affects more than 1.6 million women worldwide and takes more than 400,000 lives every year [1]. Patients with local disease have a significantly better 5 year overall survival (OS) (98%) than patients with lymph node (LN) metastasis (83.6%) or distant metastasis (23%). LN and distant metastases are therefore strong predictors of poor prognosis [2] [3]. Determination of LN status involves identification and excision of the sentinel lymph node(s) and subsequent histological examination which may include assessment of multiple levels with or without immunohistochemical (IHC) staining [4] [5]. It is recognized that about 5% of patients determined to be LN negative in fact have metastatic disease in the LN at the time of diagnosis [6] [7]. The clinical challenge is to correctly identify those patients that have or will develop LN or distant metastasis and that therefore will behave poorly, and use this information to offer supplemental treatment after local therapy. A sensitive and specific biomarker able to accurately predict disease recurrence and LN or distant metastasis is lacking and markers of disease progression continue to be needed to improve patient classification. Biomarkers such as CA 15-3 and CEA are employed to monitor the progress of the disease as an indirect measurement of tumour burden but with somewhat limited success and are not currently widely used in clinical practice [8] [9].

Differential proteomics can be used to differentiate between two physiologic states using tissue samples that represent underlying biology and pathology. Isotopic labelling [10] and label-free mass spectrometry (MS) [11] proteomics enable the quantification of proteins and thus allow direct comparison of protein expression between two sample sets [12] [13].

When organisms are exposed to harsh conditions a number of changes occur at the cellular level including changes in the secondary and tertiary structure of proteins which can lead to alterations in a protein's solubility, transport or ability to carry out its function or to perform it efficiently. Stressors that can alter protein structure include low glucose, hypoxia and acidic conditions, which are commonly seen in tumour microenvironments. One of many responses that take place when cells face stressful microenvironments is a rapid and transient increase in the expression of heat shock genes. Heat shock proteins, Heat shock protein 90 kDa beta (Grp94) member 1 (HSP90B1) being one of them, facilitate cell survival by stabilizing and refolding denatured proteins after stress [14]. HSP90B1 also helps cells escape apoptosis and preserves the function of various proto-oncogenes important for breast cancer growth [15]. HSP90 proteins have several client proteins including mutated p53 and B-RAF, BCR-ABL, v-Src, ErbB-2, AKT, RAF-1, CDK4, VEGF and PIK3 [16] [17]. The HSP90 family is comprised of 17 genes. Six have been recognized as functional in humans and these are divided into two groups: HSP90A (alpha), which includes HSP90AA1, HSP90AA2, HSP90N and HSP90AB1, and HSP90B (beta), to which HSP90B1 and TRAP1 (a.k.a HSP90B2P) belong. HSP90B is the major form of HSP90 involved in normal cellular functions, such as maintenance of the cytoarchitecture, differentiation and cytoprotection [18] [19]. Although each subgroup has slightly different characteristic functions, their functions do overlap and it is accepted that cell proliferation and differentiation are regulated by both HSP90A and HSP90B [20]. HSP90B1 has 2 known splice variants HSP90B1-201 and -202; data is lacking as to any functional difference between the two splice variants.

The purpose of this study was to use a systematic and objective method to identify protein biomarkers with possible prognostic value in breast cancer patients, particularly in discriminating cases most likely to have LN metastasis. Differential proteomic analyses were conducted on whole tissue protein extracts of cancerous and normal tissue from breast cancer patients identifying two candidate biomarkers. These were subsequently validated using prognostic tissue microarrays (TMAs).

## Materials and Methods

### Mass spectrometry Study Populations

Following University Health Network (UHN) Research Ethics Board approval, frozen tumour tissue samples from nineteen breast cancer patients (estrogen receptor (ER) positive and HER2 receptor negative) were identified and collected from the UHN BioBank. Normal samples from tissues adjacent to the tumour were collected from thirteen of nineteen patients. Cancer tissues were assigned to two groups based on axillary LN status and the thirteen normal tissue samples were used as controls for protein quantification.

### Tissue Sample Processing and Preparation

Individual tissue samples were thawed and washed three times with 1 ml of a phosphate-buffered saline (PBS) protease inhibitor cocktail (Sigma-Aldrich, St. Louis, MO) and homogenized. Equal amounts of protein were pooled from homogenized normal tissue samples to form a universal normal control sample. 100 µg of protein from each of the nineteen tumour tissue samples and the universal control were trypsinized and labeled with an iTRAQ tag as per manufacturer's instructions (Applied Biosystems, Foster City, CA).

Trypsin digested and labeled samples were randomly assigned to six 4-plex iTRAQ sets for LC-MS/MS analysis, with each set consisting of the universal control and three tumour samples (either two LN-negative samples and one LN-positive sample or vice versa).

Each set of four iTRAQ labeled samples was pooled prior to fractionation by strong cation exchange (SCX) chromatography using an Agilent 1100 HPLC system (Santa Clara, CA) coupled to a 15 cm long, 2.1 mm internal diameter Thermo BioBasic SCX column (ThermoFisher Scientific, Waltham, MA). Fractionation resulted in 30 SCX fractions per iTRAQ set, each of which was dried with a Thermo Savant SC110A speed vacuum (Holbrook, NY) and resuspended in 30 µL of 0.1% formic acid.

Stable isotope dilution (SID) experiments were performed using spike-ins of six isotope-enriched peptides in all tissue samples and analysis by selected reaction monitoring mass spectrometry (SRM-MS). Peptides were obtained from Biomatik Corporation (Canada) for HSP90B1 (P14625), 40S ribosomal protein S25 (P62851), hemoglobin subunit alpha (P69905) and alpha actin cardiac muscle 1 (P68032), additional peptides were obtained from Thermo Scientific (USA) for GTP binding nuclear protein RAN (P62826) and 14-3-3 protein zeta/delta (P63104).

### Quadrupole Time-of-flight Mass Spectrometry

SCX fractions 6 through 25 were analyzed by nano LC-MS/MS. A Proxeon nano-HPLC system (Odense, Denmark) was coupled to a QSTAR Elite Q-q-TOF mass spectrometer (AB SCIEX, USA). An information dependant data acquisition experiment was carried out with the following parameters: 250 or 500 millisecond TOF MS scan of *m/z* 400 to *m/z* 1500, MS/MS triggered on ions greater than *m/z* 400 and less than *m/z* 1500 with charge state 2 to 4 that exceeded 50 counts, former precursors excluded for 180 seconds, one survey scan and three MS/MS scans per cycle, 50 mDa mass tolerance, automatic collision energy and automatic MS/MS accumulation with a maximum accumulation of 2 seconds and a fragment intensity multiplier of 2. The second quadrupole (Q2) was manually set up with parameters optimal for sequencing and iTRAQ quantification. Data acquisition was conducted using Analyst QS 2.0 software (AB SCIEX, USA).

### Analysis of iTRAQ Dataset

Protein identification and relative quantification analyses were conducted on iTRAQ data using ProteinPilot 2.01 software (AB SCIEX, USA) based on the Paragon algorithm [21], using the following parameters: 4-plex iTRAQ, MMTS (Cys alkylation), trypsin, post-translational modifications including multiple phosphorylations, glycosylation, and other post-translational modifications due to sample processing; 66% minimum identification confidence score. Absolute fold-changes (AFC) were derived for each protein in each of the iTRAQ sets for 3 comparisons: LN negative vs LN positive; LN negative vs normal and LN positive vs normal. Requirements for putative differential expression were: LN negative vs LN positive AFC≥1.5 (113 proteins); or AFC≥1.5 in any two comparisons (189 proteins); or AFC≥3.0 in any one comparison (12 proteins).

### Selected Reaction Monitoring Mass Spectrometry

Specific SRM peptide precursor/product ion transitions were designed for each protein based upon iTRAQ results using MIDAS (MRM-initiated detection and sequencing) workflow designer software (AB SCIEX, USA). Proteins were multiplexed in sets of 50 for each SRM analysis of a patient sample using an Eksigent nano-LC coupled to a 4000 QTRAP hybrid linear ion trap/triple quadrupole mass spectrometer (AB SCIEX, USA) through a nanoflow electrospray ionization source equipped with a 15 µm ID emittor tip.

### Analysis of SRM Dataset

SRM raw data were pre-processed using MultiQuant 1.0 software (AB SCIEX, USA) to identify and derive peak areas for SRM transitions. A 2-point Gaussian smoothing procedure was applied to all peaks. Mean peak areas for each transition was compared between LN negative and LN positive groups using a test assuming unequal variance at a two-tailed significance (alpha) threshold of 0.10. Analyses were conducted using R software.

### In-House Human Breast Cancer Tissue Microarrays and Immunohistochemistry

Tissue microarrays (TMAs) were constructed at UHN from samples obtained from primary breast cancer patients admitted to Princess Margaret Hospital between January and December of 2006. For all TMAs, 4 *µm* formalin-fixed, paraffin embedded (FFPE) sections were dewaxed in five changes of xylene and brought down to water through graded alcohols. Tissue sections were microwaved in Tris-EDTA Buffer (pH 9.0) for antigen retrieval.

After blocking for 15 minutes, sections were incubated at room temperature overnight with the appropriate primary antibodies using previously optimized dilutions. Primary antibody incubation was followed by incubation with a biotinylated secondary antibody (Vector Laboratories, Burlingame, CA) for 30 minutes and horseradish peroxidase-conjugated ultrastreptavidin labeling reagent (ID Labs Biotechnology Inc., London, ON) for 30 minutes.

Stained TMA slides were scanned using a high-resolution bright field ScanScope XT scanner (Aperio Technologies, Vista, CA) at the Advanced Optical Microscopy Facility (AOMF) in the Ontario Cancer Institute (OCI). Evaluation of immunohistochemical staining was conducted using image analysis with Aperio ImageScope Software Version 9.0 (Aperio Technologies).

Statistical analysis was conducted using SPSS (Version 17.0 for Windows) software package (SPSS Inc., Chicago, IL).

### Prognostic Tissue-Microarrays, Immunohistochemistry and Scoring

Duplicate sets of Stage I and II prognostic human breast cancer TMAs were obtained from the National Cancer Institute (NCI) Cancer Diagnosis Program (Silver Spring, MD), consisting of a total of 990 (590 Stage I and 400 Stage II) distinct invasive breast cancer cases. Details for the TMAs set may be found at http://cdp.nci.nih.gov/breast/prognostic_cs.html.

A summary of patients' clinical and pathological characteristics for the TMAs is provided in Table S1. Immunostaining was performed according to standard protocols using one of the following primary antibodies: Decorin (HPA003315 - Sigma-Aldrich, St. Louis, MO) at a concentration of 1∶400 overnight, HSP90B1 (HPA003901 - Sigma-Aldrich, St. Louis, MO) at 1∶4000 for 1 hour, Ki-67 (SP6, LabVision, Fremont, CA) at 1∶1000 for 1 hour, ER (SP1, LabVision) at 1∶200 for 1 hour, and HER2 (4B5, Ventana, Tucson, AZ) at 1∶50 for 1 hour. A summary of marker characteristics for DCN and HSP90B1 is provided in Table S2.

**Figure 1:**
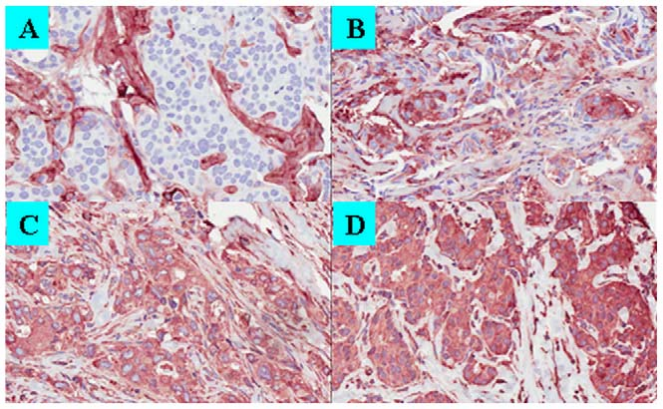
Scoring system used for DCN and HSP90B1 immunohistochemistry. A, Strong DCN positivity in stroma (3+) and negative in carcinoma (0) (magnification 200×). B, Strong DCN positivity in carcinoma (3+), weak stromal positivity (1+) (200×). C, Moderate HSP90B1 positivity in carcinoma (1+) (200×). D, Strong HSP90B1 positivity in carcinoma (3+) (200×). Decorin antibody (Sigma-Aldrich, St. Louis, MO) used at a dilution of 1∶400. HSP90B1 antibody (Sigma-Aldrich, St. Louis, MO) used at a dilution of 1∶4000.

Tissue microarray IHC staining was evaluated under light microscopy and with software-based image analysis (Aperio Technologies, Vista, CA,). Decorin staining intensity was assessed in both normal stromal cells and cancer epithelial cells separately under light microscopy, HSP90B1 staining was scored in the invasive cancer only. The average staining intensity of DCN (Decorin_Iavg) and HSP90B1 (HSP90B1_Iavg) was quantified using Aperio image analysis. TMAs were analyzed blindly and independently by two pathologists (DTT and JM) using a four point semi-quantitative scale for intensity: 3+ (very strong), 2+ (strong), 1+ (moderate/weak), and 0 (no staining) (Figure 1). In case of disagreement cores were reviewed together and consensus was reached. TMAs were scanned at 20× and the ImageScope Positive Pixel Count algorithm version 9.1 used for software-based analysis on the entire core. An average intensity of positive pixels is calculated by the software generating a continuous variable of intensity scores in which higher scores (pixel colour closer to white) mean lower staining intensity.

Given the heterogeneity and relatively low DCN epithelial staining, any strong even if focal, cancer cell staining was considered high expression, while due to the more diffuse moderate to strong HSP90B1 positivity in the majority of cores, we required both diffuse and strong HSP90B1 positivity to categorize it as high expression. For analytical purposes, 3+ and 2+ staining were considered high expression for both DCN and HSP90B1; and 1+ and 0 staining were considered low expression. Evaluation of ER and HER2 staining was performed using current recommendations [22], [23]. HER2 cases that were considered equivocal (2+) were omitted from the analysis since fluorescence in situ hybridization staining for HER2 was not available. Breast cancer molecular subtypes were defined by IHC expression of ER, and Ki-67 as suggested by Cheang et al and Hugh et al: Luminal A (ER-positive, HER2-negative and low Ki67), luminal B (ER-positive, HER2-positive and/or high Ki67), HER2 (ER-negative and HER2-positive) and basal-like (ER-negative and HER2-negative) [24] [25].

### Statistical Analysis of TMA Datasets

The association between demographic and clinicopathological variables and the dichotomized values for DCN staining in stroma and malignant tissue as well as HSP90B1 staining in tumour tissues was evaluated using the Chi-square test for categorical factors. Decorin_Iavg and HSP90B1_Iavg variables had a normal distribution. The association between patient clinicopathological variables and the continuous marker parameters Decorin_Iavg and HSP90B1_Iavg were assessed using a two-sided t test or ANOVA, as appropriate. Kaplan-Meier survival curves were constructed for disease-free survival (DFS) and OS and differences between groups were determined using the log-rank test. The prognostic value of the dichotomized and continuous marker parameters was evaluated using univariate and multivariable Cox proportional hazard models, adjusting for clinicopathological variables. All statistical analyses were performed using SAS 9.2 (SAS Institute, Cary, NC). All reported p values are two-sided and a value <0.05 was considered statistically significant. This study has been designed and reported following the “Reporting recommendations for tumour marker prognostic studies (REMARK)” guidelines [26].

## Results

**Figure 2:**
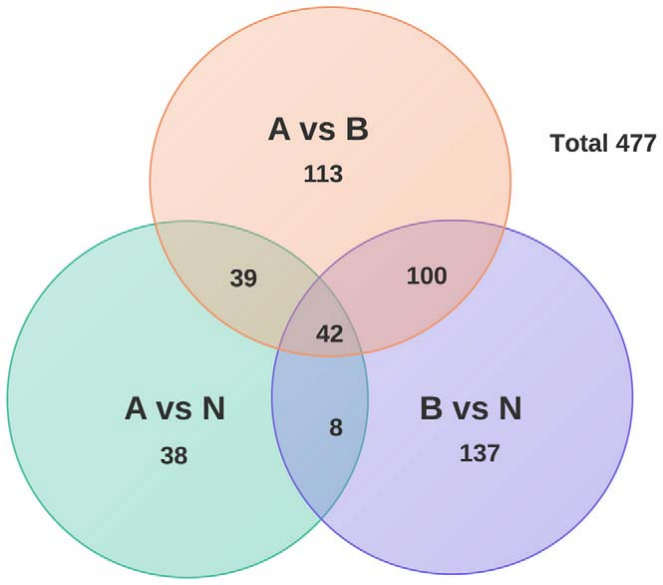
Venn diagram of Differentially expressed proteins. Number of proteins differentially expressed for each comparison group as well as overlapping proteins. Node-negative breast cancer tissue (group A), node positive BC tissue (group B), and normal breast tissue (group N).

Quantitative proteomic profiling using iTRAQ-labelling identified 988 proteins of which a subset of 477 were determined to be differentially expressed between LN positive and LN negative cancer tissues or between cancer and normal tissues based on a minimum absolute fold-change of 1.5 (Figure 2). The ProteinPilot search results of the iTRAQ experiments at the protein level are presented in Table S3. Differential expression was verified by targeted quantification using label-free and SID SRM-MS [11] on breast tumour tissue. Over 70% of the significant proteins identified by iTRAQ-MS were detected by label-free SRM-MS, and differential expression of 49 proteins was confirmed (18 p<0.05 and 31 0.05<p<0.10), of which 23 displayed increased expression and, 26 displayed decreased expression in LN positive tissues (Table S4). Box plots summarizing these results are shown in (Data S1). Further, SID SRM-MS using peptides from HSP90B1 (P14625), 40S ribosomal protein S25 (P62851), hemoglobin subunit alpha (P69905), alpha actin cardiac muscle 1 (P68032), GTP binding nuclear protein RAN (P62826) and 30 additional 14-3-3 protein zeta/delta (P63104) confirmed the identification of all except RAN (Table 1).

**Table 1:** Confirmation of peptide identity using SID SRM-MS.

Name	Accession	Peptide used for SID	Confirmed
Endoplasmin precursor	P14625	SILFVPTSAPR	Yes
40S ribosomal protein S25	P62851	LITPAVVSER	Yes
Hemoglobin subunit alpha	P69905	LRVDPVNFK	Yes
Actin, alpha cardiac muscle 1	P68032	SYELPDGQVITIGNER	Yes
GTP-binding nuclear protein RAN	P62826	AAQGEPQVQFK	No
14-3-3 protein zeta/delta	P63104	NLLSVAYK	Yes

List of peptides for which SID SRM-MS was used to confirm their identification. UniProtKB accession number and amino acid sequence are shown.

SID: Stable isotope dilution.

SRM-MS: selected reaction monitoring mass spectrometry.

Ten proteins with commercially available antibodies were screened on one tissue microarray (TMA) of invasive ductal carcinoma (Table 2). Five candidates (HSP90B1, HMGN2, USP34, DCN and G6PD) showed a tentative association with LN status. HSP90B1 and DCN were positively correlated and USP34, G6PD and HMGN2 were negatively correlated with axillary LN status.

**Table 2:** Antibodies used to validate putative biomarkers.

Antibody Name	Laboratory
Lumican precursor (LUM)	Sigma-Aldrich
Endoplasmin (HSP90B1)	Sigma-Aldrich
Alpha-2-HS-glycoprotein precursor (AHSG)	Sigma-Aldrich
60-kDa heat shock protein mitochondrial precursor (HSPD1)	Sigma-Aldrich
Ubiquitin carboxyl-terminal hydrolase 34 (USP34)	Sigma-Aldrich
Decorin precursor (DCN)	Sigma-Aldrich
Ceruloplasmin precursor (CP)	Sigma-Aldrich
Glucose-6-phosphate-1-dehydrogenase (G6PD)	Sigma-Aldrich
Nonhistone chromosomal protein HMG-17 (HMGN2)	Lifespan Biosciences
haptoglobin precursor (HP)	Lifespan Biosciences

List of commercially available antibodies purchased, including manufacturing laboratory, used to validate putative biomarkers. Only antibodies designed for use on formalin fixed paraffin embedded tissue were used.

Mean TMA protein expression levels of HSP90B1 and DCN showed the strongest statistical association with presence of LN metastasis being significantly higher in LN positive tumours relative to LN negative tumours (HSP90B1 p = 0.049; DCN p<0.001). Subset analysis of ER-positive and HER2-negative cases revealed a greater mean expression difference for both HSP90B1 and DCN. The expressions of HSP90B1, USP34 and HMGN2 were significantly negatively associated with tumour grade (p<0.001).

### High DCN and HSP90B1 Predict Metastasis and Worse OS

Following the MS results the expression levels of DCN and HSP90B1 and their association with clinicopathological characteristics were further assessed in breast cancer tissues from an independent cohort compiled by the NCI and distributed as breast cancer prognostic TMAs.

Cytoplasmic staining was seen for both DCN and HSP90B1. Of the 990 cases 928 (94%), 967 (98%) and 930 (94%) were interpretable for DCN staining in stroma, DCN staining in the carcinoma and HSP90B1 staining in the carcinoma, respectively. High expression of DCN in stroma, carcinoma and HSP90B1 staining in carcinoma was seen in 76%, 34% and 84% of cases, respectively (see Table S2).

High DCN expression in stroma correlated with lower tumour grade (p<0.0001), Ki67 level ≤10% (p = 0.005) and ER positivity (p = 0.0002). These correlations were also found with lower levels of software-based scoring Decorin_Iavg, (all p<0.001). No correlation was found between intensity of DCN staining in stroma and LN status.

High expression of DCN in the malignant epithelium was correlated with LN positivity (p<0.001), higher number of positive lymph nodes (p<0.0001) and HER2-positive status (p = 0.004) compared to patients with low expression. Subgroup analysis by molecular type showed similar odds ratios (OR) for this correlation in 3 out of the 4, subgroups: luminal A (OR: 2.34), luminal B (OR: 2.39) and HER2 (OR: 2.39), respectively.

Patients with high expression of HSP90B1 in malignant cells were more likely to have distant metastasis compared to patients with low expression (17% vs. 3%, p = 0.0002). This correlation was also found with lower levels (i.e. higher staining intensity) of software-based scoring HSP90B1_Iavg (p = 0.03). Lower levels of HSP90B1_Iavg were also associated with tumour size ≤2 cm (p<0.0001), lower grade (p = 0.001), negative LN status (p<0.0001), Ki67≤10 (p = 0.002) and ER positivity (p = 0.002)). Performance analysis revealed that high DCN expression in the malignant tissue predicts LN metastasis with a sensitivity of 48% and a specificity of 70.8%. High HSP90B1 expression in malignant epithelial cells predicts distant metastasis with a sensitivity of 97.1% and a specificity of 14.2%.

Univariate Cox proportional hazards regression analysis found higher staining of the malignant epithelial tissue with DCN and HSP90B1 to be predictors of decreased OS with a HR equal to 1.29 (p = 0.01) and 2.12 (p<0.0001), respectively. Age, tumour size, tumour grade and LN status were also associated with OS. All of these variables retained significance on multivariate analysis (Table 3). Continuous markers (Decorin_Iavg and HSP90B1_Iavg) were not found to be predictors of OS or DFS (DFS data not shown).

**Table 3:** Univariate and multivariate analysis of predictors of overall survival.

		Overall Survival			
		Univariate		Multivariate (N = 864)	
	Characteristic	Unadjusted HR (95% CI)	p	Adjusted HR (95% CI)	p
Age	≤50	1.00	<0.0001	1.00	<0.0001
	>50	1.99 (1.54–2.57)		2.73 (2.04–3.66)	
Tumour Size	≤2 cm	1.00	<0.0001	1.00	<0.0001
	>2 cm	1.83 (1.50–2.24)		1.59 (1.26–2.01)	
Tumour Grade	I	1.00	0.002	1.00	0.007
	II	1.32 (1.04–1.67)		1.29 (0.66–1.67)	
	III	1.58 (1.23–2.04)		1.75 (1.23–2.47)	
LN Status	Positive	1.00	<0.0001	1.00	0.001
	Negative	1.63 (1.33–2.00)		1.50 (1.18–1.90)	
Ki 67	≤10	1.00	0.4	1.00	0.17
	>10	1.10 (0.88–1.39)		0.76 (0.51–1.13)	
Molecular Subtype	Luminal A	1.00	0.49	1.00	0.59
	Luminal B	1.19 (0.89–1.60)		1.35 (0.86–2.12)	
	Her2	1.25 (0.81–1.91)		1.04 (0.64–1.69)	
	Basal	1.12 (0.84–1.50)		1.25 (0.82–1.90)	
Decorin Stroma	Low	1.00	0.06		0.01
	High	1.28 (0.99–1.65)		1.55 (1.11–2.17)	
Decorin Epithelium	Low	1.00	0.01	1.00	0.05
	High	1.29 (1.06–1.57)		1.25 (0.998–1.55)	
Decorin_avg		1.00 (0.997–1.006)	0.59		
Decorin_Iwavg		1.00 (0.995–1.01)	0.49		
HSP90B1 Epithelium	Low	1.00	<0.0001	1.00	<0.0001
	High	2.12 (1.48–3.02)		2.16 (1.49–3.14)	
HSP90B1_Iavg		1.00 (0.996–1.008)	0.46	1.00 (0.996–1.01)	0.39

**Univariate and Multivariable Cox proportional hazards regression to determine predictors of overall survival. Note: Molecular subtype was used in place of ER and HER2 status; Decorin Iwavg was used in place of Decorin Iavg.**

**Figure 3:**
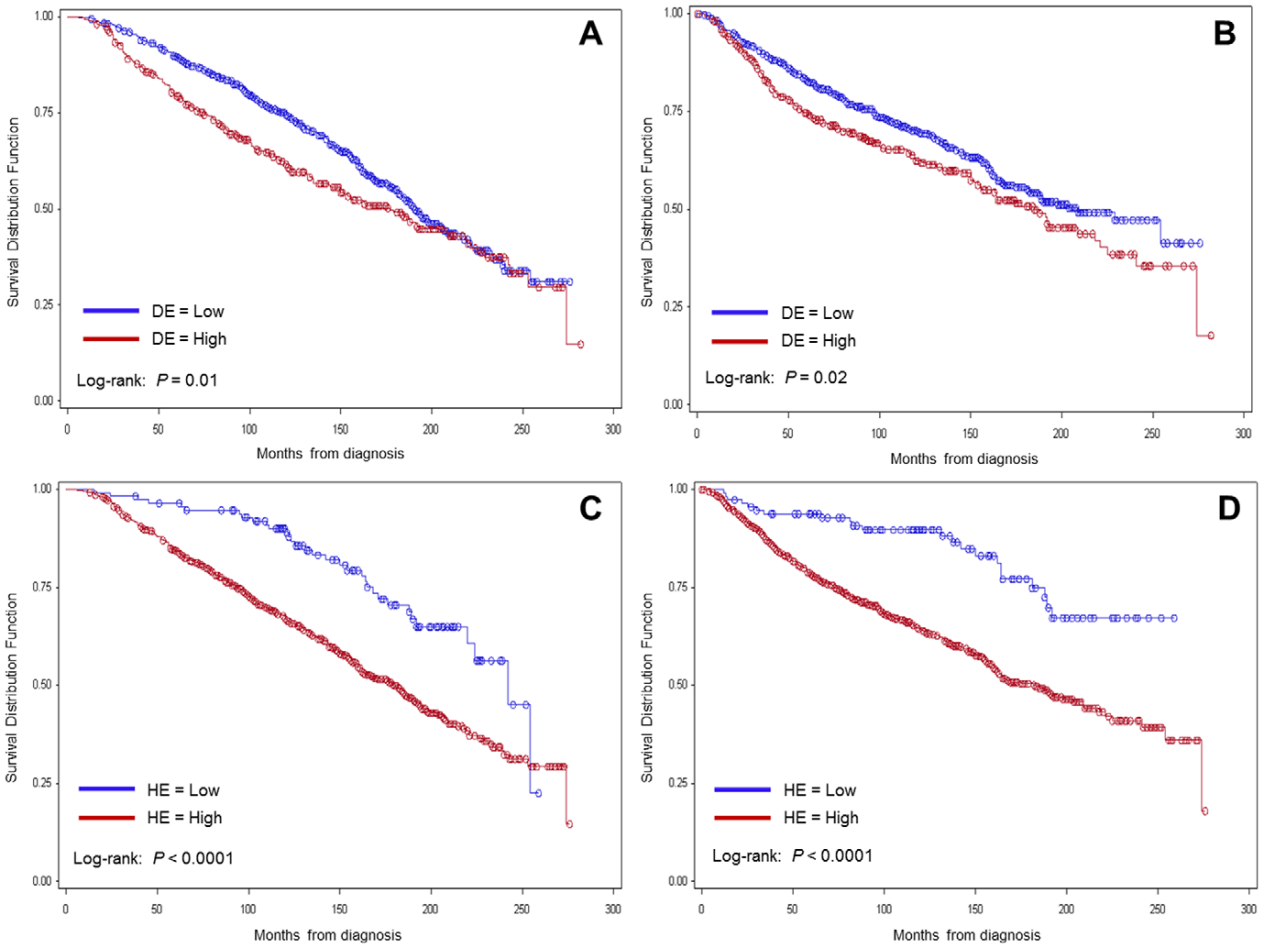
Overall and Disease-free survival based on high and low DCN and HSP90B1 staining. A, OS curve for DE. B, DFS curve for DE. C, OS curve for HE. D, DFS curve for HE. DE: Decorin staining in malignant epithelial tissue. HE: HSP90B1 staining in malignant epithelial tissue. OS: Time from diagnosis to death from any cause. DFS: Time from diagnosis to any recurrence or death from any cause.

**Figure 4:**
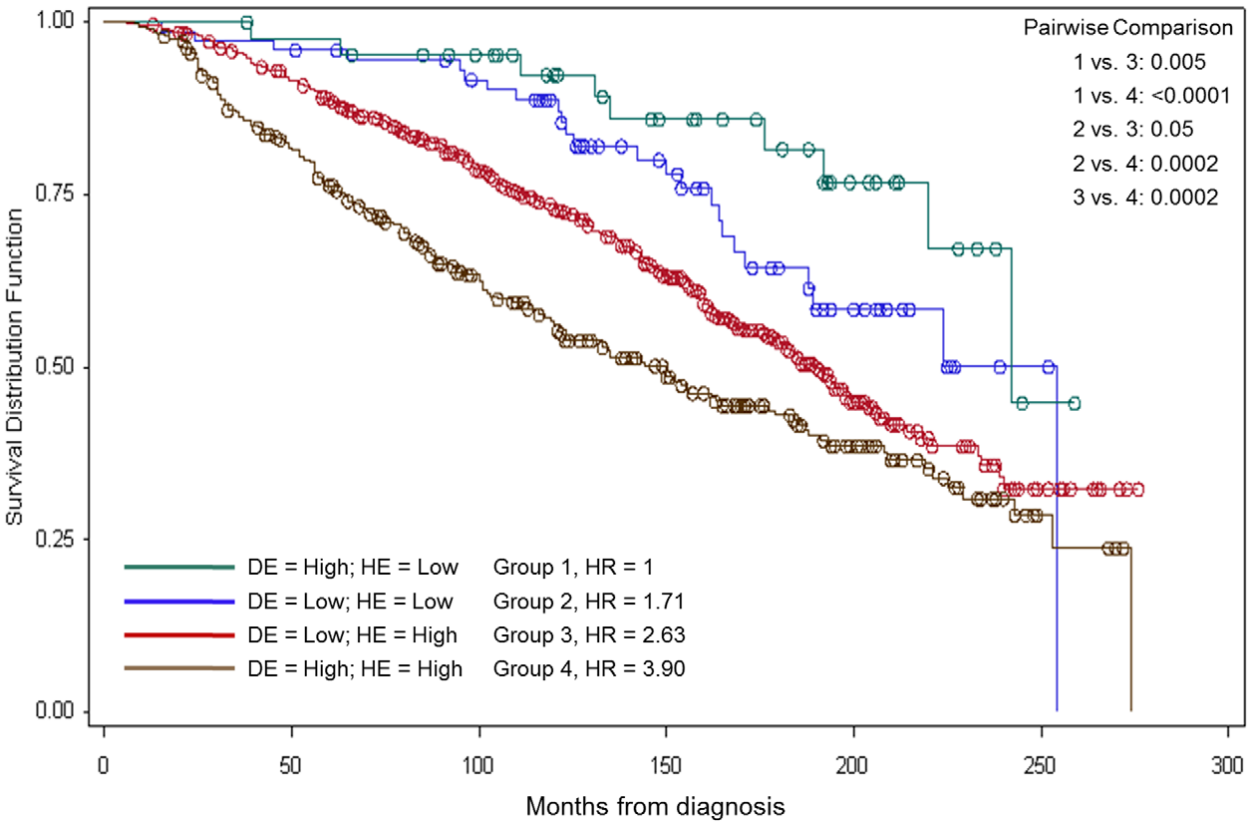
Overall survival curves using combinations of DE and HE expression levels. Univariate Cox regression used to determine HR; logrank p-values reported; Bonferroni multiple testing adjustment for pairwise comparisons p = 0.05/5 = 0.01. DE: Decorin staining in malignant epithelial tissue. HE: HSP90B1 staining in malignant epithelial tissue. HR: Hazard ratio.

Kaplan-Meier analysis (median follow-up of 12.8 years, range: 1.1 to 23.5 years) showed that patients whose tumours express high levels of DCN or HSP90B1 in the malignant epithelium have significantly lower OS and DFS compared to patients with lower expression of either marker (Figure 3). Combination group analysis showed that patients with high expression of both markers have the worse OS (p<0.0001) (Figure 4) and DFS (p<0.0001) of all four groups.

**Figure 5:**
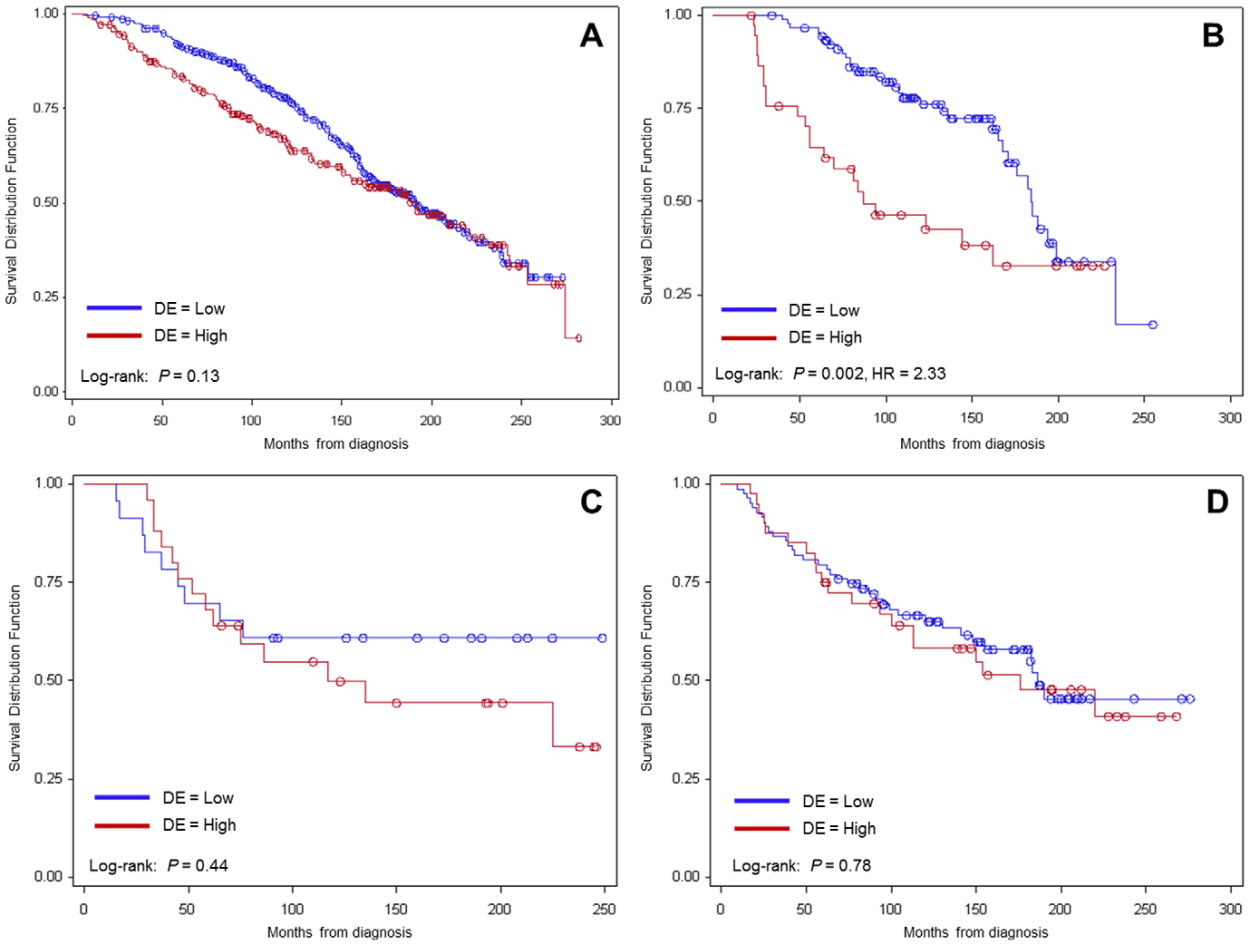
Overall survival curves for high and low DE staining based on tumour molecular subtype. Univariate Cox regression used to determine HR; logrank p-values reported. Molecular subtypes were defined by IHC expression of ER, HER2 and Ki-67 as suggested by Cheang *et al.* (2009) and Hugh *et al.* (2009). DE: Decorin staining in malignant epithelial tissue. HR: Hazard ratio.

**Figure 6:**
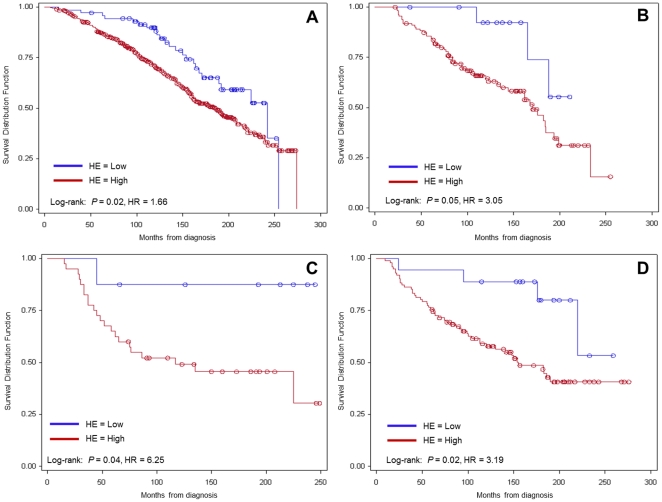
Overall survival curves for high and low HE staining based on tumour molecular subtype. Univariate Cox regression used to determine HR; logrank p-values reported. Molecular subtypes were defined by IHC expression of ER, HER2 and Ki-67 as suggested by Cheang *et al.* (2009) and Hugh *et al.* (2009). HE: HSP90B1 staining in malignant epithelial tissue. HR: Hazard ratio.

Molecular subtype analysis indicated that high expression of DCN in malignant epithelial cells is a predictor of decreased OS (HR: 2.33 vs. low expression p = 0.002) only in luminal B subtype tumours (Figure 5) as is high expression of DCN in the benign peri-lesional stroma (data not shown). High expression of HSP90B1 in malignant epithelial cells is associated with lower OS in all four groups: Luminal A (HR: 1.66 vs. low expression, p = 0.02), Luminal B (HR: 3.05 p = 0.05), HER2 (HR: 6.25, p = 0.04) and basal subtype (HR: 3.19, p = 0.02) (Figure 6). This was also the case for DFS (data not shown).

**Figure 7:**
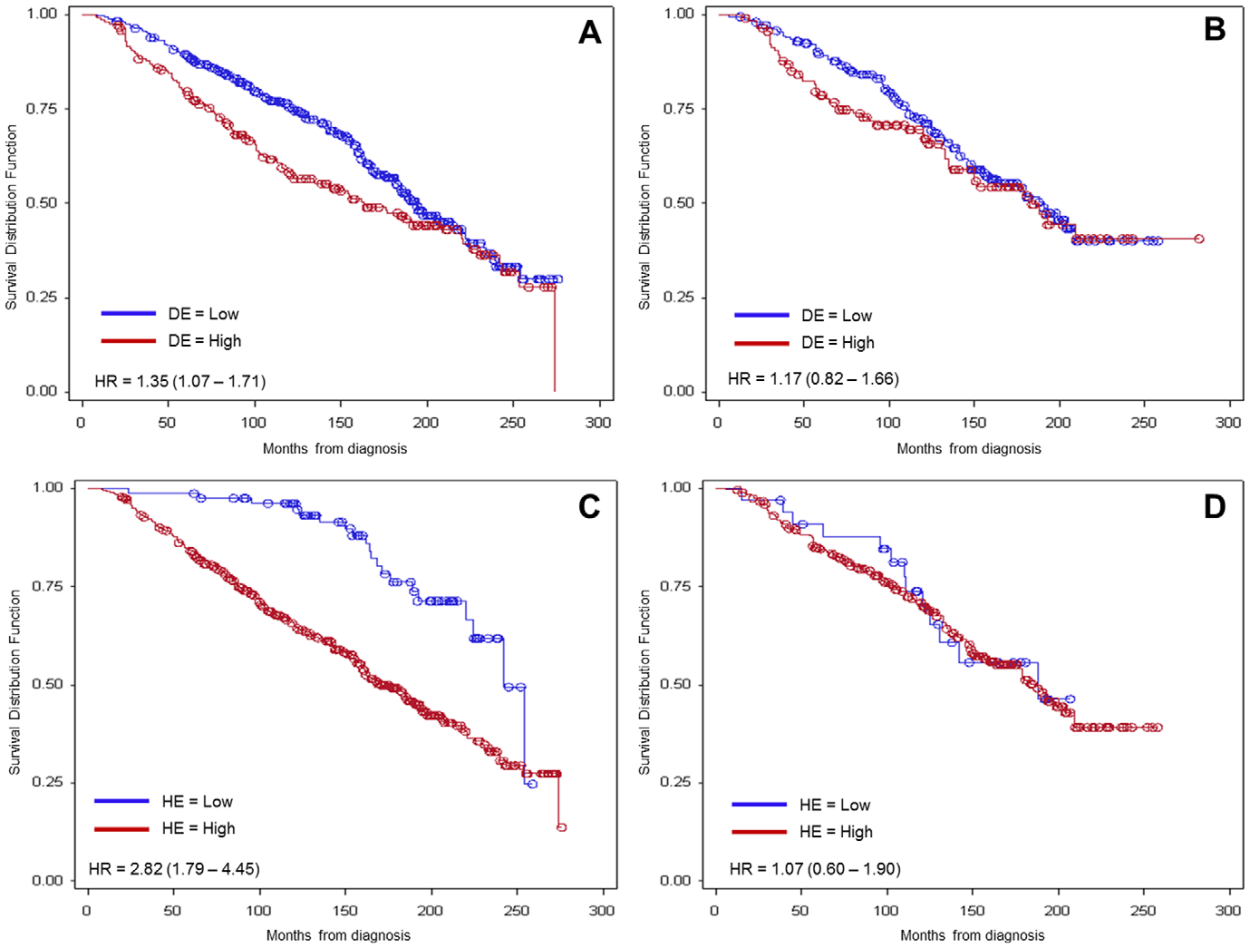
Overall survival curves based on DE/HE expression and hormone treatment. A, Survival curves for cases with high and low DE that did not receive hormone treatment. B, Survival curves for cases with high and low DE that received hormone treatment. C, Survival curves for cases with high and low HE that did not receive hormone treatment. D, Survival curves for cases with high and low HE that received hormone treatment. Univariate Cox regression used to determine HR and 95% CI. DE: Decorin staining in malignant epithelial tissue. HE: HSP90B1 staining in malignant epithelial tissue.

Survival analysis based on hormone treatment group showed that OS of patients in which malignant epithelial cells have high expression of DCN or HSP90B1 benefited significantly from hormone treatment (Figure 7), with a HR after hormone treatment approaching that of patients with low expression of both markers. Chemotherapy did not change OS in either group.

## Discussion

The purpose of this study was to use a systematic and objective method to identify possible biomarkers that could have prognostic value in breast cancer patients, particularly in identifying cases most likely to have LN metastasis. We performed differential proteomic analyses of whole tissue protein extracts of cancerous and normal tissue from breast cancer patients. The initial discovery phase combined iTRAQ labelling with off-line two-dimensional liquid chromatography tandem MS, for global, unbiased protein profiling and quantification. Subsequently label-free SRM-MS was used for targeted quantification of differentially expressed proteins to verify differential expression in individual tissue samples. In the end, parallel, isotope enriched peptides for 6 significant proteins identified by iTRAQ-MS were synthesized for SID SRM-MS analysis of individual tissue samples, providing confirmation of identification for 5 proteins, including HSP90B1. TMA analysis revealed that the expression levels of two candidate markers were positively associated with LN metastasis: DCN (p = 0.001) and HSP90B1 (p = 0.049) Finally, IHC analysis using the NCI prognostic TMAs showed significant association of high expression of DCN with LN metastasis, high expression of HSP90B1 with distant metastasis and high expression of both markers with decreased OS and DFS.

DCN and HSP90B1 play important roles in several biological pathways related to tumorigenesis. Decorin is a key modulator of the tumour microenvironment [27] through interactions with EGFR and MAPK [28] pathways. Decorin also activates insulin-like growth factor-I receptor [29], attenuates Erb2 signalling [30], binds to TGF-Beta, activates Met and up-regulates p21 [31] [32]. While in most studies DCN has been found to have an antioncogenic role, others correlate DCN with increased migration of human osteosarcoma cells [33] and high expression in endothelial cells undergoing angiogenesis [34]. HSP90B1 is a heat shock chaperone protein that stabilizes and refolds denatured proteins after stress, facilitating cell survival during conditions commonly seen in the tumour microenvironment [33]. HSP90 proteins are involved in the glucocorticoid receptor and the AKT signalling pathways [35] [36], through these interactions they increase glucose metabolism, cell proliferation, transcription and cell migration and decreased apoptosis. HSP90 proteins have been found increased in metastatic melanoma compared to the primary [37] and high HSP90 expression predicts worse OS in patients with acute lymphocytic leukemia [38] and breast cancer [39], and decreased DFS in gastrointestinal stromal tumours [40]. Several phase II and III trials are evaluating the anticancer activity of HSP90 inhibitors in several types of cancer.

The MS findings suggested that high DCN and high HSP90B1 expression were associated with LN metastasis, with SRM-MS based fold-change of 1.5 observed for DCN (p = 0.06) and 2.0 for HSP90B1 (p = 0.007) in LN positive tissues. This association was confirmed for DCN staining of epithelial cancer cells (p<0.001), which was also associated with the number of positive LNs (p<0.0001) and worse OS. It is interesting, albeit unexplained, to find that in our study the negative prognostic value for survival in patients with high DCN expression in malignant cells was clearly significant in Luminal B cases (i.e. ER-positive, HER2-positive) (HR: 2.33 p = 0.002) while lacking any survival prognostic effect on ER only or HER2 only positive tumours. Nevertheless, a marker of LN metastasis risk and worse OS risk exclusive to this subgroup of patients could have clinical utility.

The expected association between high HSP90B1 expression and LN metastasis was not seen, however a significant association between high HSP90B1 expression and distant metastasis was found (median follow-up 11.5 years, p<0.0001 for dichotomous variable HSP90B1 epithelium and p = 0.03 for continuous variable HSP90B1_Iavg). Given that vascular endothelial growth factor-A, a potent pro-angiogenic factor, is down-stream of the EGFR pathway which is in turn increased by HSP90, it may be that high levels of HSP90 could promote metastasis, and in fact an association between high HSP90 expression and metastasis in melanoma has been reported previously. However to the best of our knowledge this association has not been described in breast cancer. Even more interesting is the fact that high HSP90B1 levels were also associated with parameters considered to confer good prognosis to breast cancer patients (i.e. tumour size ≤2, lower grade, negative LN status, ER positivity, Ki67≤10 and lower grade), although this association was found only with the software-based score. Taken together these findings suggest that high expression of HSP90B1 could potentially identify a subgroup of patients that, based on currently used clinicopathologic variables, are considered to have good prognosis and yet have shorter OS and DFS. OS results found in combination group analysis suggest that having high levels of HSP90B1 in malignant cells is worse that having high levels of DCN. This was also supported in the multivariate analysis as high HSP90B1 staining in malignant epithelial cells had a higher HR than high DCN staining in the same cells.

An important and novel finding is that in terms of OS, patients with high expression of HSP90B1 in tumour cells appear to benefit significantly from hormonal treatment (HR: 1.07 vs. 2.8 for no hormone treatment group). This HR is similar to that of the group with low expression of HSP90B1 in malignant epithelial cells. This hormonal treatment effect cannot be explained by hormone receptor status bias because OS was significantly better for patients with low vs. high tumour expression of HSP90B1 for all molecular subtypes. A similar hormonal treatment benefit was seen for patients with high DCN staining in malignant cells, albeit with a less dramatic improvement (HR: 1.17 vs. 1.35 for no hormone treatment group) and can be at least partially explained by molecular subtype bias since high DCN staining in malignant cells had prognostic value for OS only for Luminal B type of tumours (i.e. ER-positive/HER2 -positive).

We describe the verification of iTRAQ-based discoveries using quantitative SRM-MS in fresh frozen tissue, as well as preliminary validation of two markers using another analytical technology (i.e. IHC), a different sample preparation (FFPE), and two independent sample populations. Of the proteins showing significant differential expression between LN positive and LN negative samples based on SRM-MS, 10 had commercially available antibodies suitable for use on FFPE tissue. Despite the different technique and sample type, 5 of the 10 antibodies showed statistically significant correlation with LN status when protein expression was analyzed by IHC in an independent cohort of 39 patients (UHN). When the analysis was extended to 234 cases (UHN), DCN and HSP90B1 remained statically significant. Moreover, when prognostic TMAs (NCI) were used a significant association with decreased survival was observed. Although the verification of DCN and HSP90B1 in an independent cohort was encouraging, the other proteins identified during discovery for which commercial antibodies were available did not replicate in this assay. There are several factors that may have contributed to the differences in protein identification seen in different stages of this study. SRM-MS and IHC measure protein expression in entirely orthogonal ways, (i.e. SRM-MS detects peptides as surrogates of protein expression, while IHC detects specific epitopes of target proteins) and formalin fixation may alter protein quality (e.g. through crosslinking). Furthermore, different cohorts were used and therefore inherent differences between the two cohorts and heterogeneity of the samples are to be expected.

This study aims to present the proteomics' discovery results and their correlation with IHC focusing on their prognostic capability. We found it promising that using an inexpensive and ubiquitous technique such as IHC, we were able to corroborate that both groups (LN positive vs LN negative breast cancers) have significant differences in respect to these two proteins, and that these differences are associated with decreased overall survival. External validation of these markers using blotting or molecular techniques and separate cohorts would be the next desirable step.

As with any IHC-based study, limitations exist due to limited technical reproducibility and subjective interpretation of IHC staining. In addition, the strong ubiquitous DCN stroma staining makes it difficult to evaluate DCN epithelial staining in some invasive cancers with a single-cell invasive pattern. As well, the generally positive HSP90B1 epithelial expression forces the selection of a high cut-off point that clearly separates two groups, one with higher expression than the other.

Using proteomic profiling of primary breast cancers two new promising prognostic and predictive markers were found to discriminate patients with worse survival. In addition, high expression of HSP90B1 appears to be a marker for distant metastasis and a predictive marker for hormone therapy benefit even in patients with negative hormone receptor status. Additional research is needed to determine the clinical role for these markers.

## Supporting Information

Table S1 **Summary of clinicopathological characteristics of the NCI TMA cohort.** Summary of clinical and pathological characteristics of all cases included in the NCI prognostic TMAs. Molecular subtypes were defined by IHC expression of ER, HER2 and Ki-67 as suggested by Cheang et al. (2009) and Hugh et al. (2009). TMA: Tissue microarray. NCI: National Cancer Institute.
(DOC)

Table S2 **Marker characteristics of the entire cohort.** Summary of marker characteristics (Decorin and HSP90B1) for all the cases in the cohort (N = 967).
(DOC)

Table S3 **List of iTRAQ experiments' results at the protein level.** Protein names and accession numbers given according to UniProtKB/Swiss-Prot.
(XLS)

Table S4 **Differentially expressed proteins identified by SRM analysis.** Proteins identified by SRM-MS analysis with significant difference in mean expression (p<0.10) between node-negative and node-positive tumour tissue. Proteins displaying significance in more than one SRM transition are marked with an asterisk (*), with minimum p-value and maximum fold-change reported. SRM: Selected reaction monitoring. MS: Mass spectrometry.
(DOC)

Data S1 **Box plots summarizing peak values of proteins identified by SRM-MS analysis.** SRM-MS analysis identified 49 proteins with significant difference in mean expression (p<0.10) between node-negative and node-positive tumour tissue. SRM: Selected reaction monitoring. MS: Mass spectrometry.
(DOC)
