# Supplementary material for: Proteomic Analyses Reveal High Expression of Decorin and Endoplasmin (HSP90B1) Are Associated with Breast Cancer Metastasis and Decreased Survival
Source: PLoS One. 2012 Feb 20;7(2):e30992. doi: 10.1371/journal.pone.0030992 (PMC3282708; doi:10.1371/journal.pone.0030992)
Supplement: Table S1 — Summary of clinicopathological characteristics of the NCI TMA cohort. Summary of clinical and pathological characteristics of all cases included in the NCI prognostic TMAs. Molecular subtypes were defined by IHC expression of ER, HER2 and Ki-67 as suggested by Cheang et al. (2009) and Hugh et al. (2009). TMA: Tissue microarray. NCI: National Cancer Institute. (DOC) [file pone.0030992.s001.doc]

**Supplemental Table S1. Summary of clinicopathological characteristics of the NCI TMA cohort**

|  |  | **All patients (N=967)** | |
| --- | --- | --- | --- |
| **Characteristic** | | N | % |
| ***Age*** | |  |  |
|  | Median (range) | 61 (26 - 96) | |
|  | ≤ 50 | 254 | 26% |
|  | > 50 | 713 | 74% |
| ***Tumour size*** | |  |  |
|  | Median (range) | 1.6 (0.5 - 9.5) | |
|  | ≤ 2 | 704 | 73% |
|  | > 2 to ≤ 5 | 255 | 26% |
|  | > 5 | 8 | 1% |
| ***Tumour grade*** | |  |  |
|  | I | 269 | 28% |
|  | II | 443 | 46% |
|  | III | 255 | 26% |
| ***LN status*** | |  |  |
|  | Positive | 271 | 28% |
|  | Negative | 696 | 72% |
| ***Number of positive nodes*** | |  |  |
|  | Median (range) | 0 (0 - 98) | |
|  | 0 | 696 | 72% |
|  | 1 - 3 | 168 | 17% |
|  | 4 - 10 | 83 | 9% |
|  | > 10 | 20 | 2% |
| ***ER status*** | |  |  |
|  | Positive (1, 2) | 775 | 80% |
|  | Negative (0) | 180 | 19% |
|  | Unknown | 12 | 1% |
| ***HER2 status*** | |  |  |
|  | Positive (2.5, 3) | 89 | 9% |
|  | Negative (0, 1) | 834 | 86% |
|  | Unknown | 44 | 5% |
| ***Ki67*** | |  |  |
|  | Median (range) | 7.5 (0 - 80) | |
|  | ≤ 10 | 746 | 77% |
|  | > 10 | 211 | 22% |
|  | Unknown | 10 | 1% |
| ***Molecular subtype*** | |  |  |
|  | Luminal A | 614 | 63% |
|  | Luminal B | 129 | 13% |
|  | HER2 | 48 | 5% |
|  | Basal | 123 | 13% |
|  | Unknown | 53 | 5% |
| ***Chemotherapy*** | |  |  |
|  | Yes | 256 | 26% |
|  | No | 711 | 74% |
| ***Radiation therapy*** | |  |  |
|  | Yes | 328 | 34% |
|  | No | 639 | 66% |
| ***Hormone therapy*** | |  |  |
|  | Yes | 334 | 35% |
|  | No | 633 | 65% |

Summary of clinical and pathological characteristics of all cases included in the NCI prognostic TMAs. Molecular subtypes were defined by IHC expression of ER, HER2 and Ki-67 as suggested by Cheang et al. (2009) and Hugh et al. (2009).

TMA: Tissue microarray

NCI: National Cancer Institute
