# Supplementary material for: Proteomic Analyses Reveal High Expression of Decorin and Endoplasmin (HSP90B1) Are Associated with Breast Cancer Metastasis and Decreased Survival
Source: PLoS One. 2012 Feb 20;7(2):e30992. doi: 10.1371/journal.pone.0030992 (PMC3282708; doi:10.1371/journal.pone.0030992)
Supplement: Table S2 — Marker characteristics of the entire cohort. Summary of marker characteristics (Decorin and HSP90B1) for all the cases in the cohort (N = 967). (DOC) [file pone.0030992.s002.doc]

**Supplemental Table S2. Marker characteristics of the entire cohort.**

|  |  | **All patients (N=967)** | |
| --- | --- | --- | --- |
| **Characteristic** | | N | % |
| ***Decorin Stroma*** | |  |  |
|  | High (1) | 737 | 76% |
|  | Low (0) | 191 | 20% |
|  | Unknown | 39 | 4% |
| ***Decorin Epithelium*** | |  |  |
|  | High (1) | 333 | 34% |
|  | Low (0) | 634 | 66% |
|  | Unknown | 0 | 0% |
| ***Decorin_Iavg*** | |  |  |
|  | Median | 148.25 | |
|  | Mean | 146.88 | |
|  | Standard deviation | 21.74 | |
|  | Range | 61.44 - 212.00 | |
|  | Unknown | N = 10 | |
| ***Decorin_Iwavg*** | |  |  |
|  | Median | 157.72 | |
|  | Mean | 158.49 | |
|  | Standard deviation | 13.16 | |
|  | Range | 122.65 - 212.00 | |
|  | Unknown | N = 10 | |
| ***HSP90B1 Epithelium*** | |  |  |
|  | High (2, 3) | 814 | 84% |
|  | Low (1) | 116 | 12% |
|  | Unknown | 37 | 4% |
| ***HSP90B1_Iavg*** | |  |  |
|  | Median | 143.72 | |
|  | Mean | 143.90 | |
|  | Standard deviation | 15.60 | |
|  | Range | 79.75 - 220.00 | |
|  | Unknown | N = 21 | |

Summary of marker characteristics (Decorin and HSP90B1) for all the cases in the cohort (N=967).
