# Supplementary material for: Proteomic Analyses Reveal High Expression of Decorin and Endoplasmin (HSP90B1) Are Associated with Breast Cancer Metastasis and Decreased Survival
Source: PLoS One. 2012 Feb 20;7(2):e30992. doi: 10.1371/journal.pone.0030992 (PMC3282708; doi:10.1371/journal.pone.0030992)
Supplement: Table S4 — Differentially expressed proteins identified by SRM analysis. Proteins identified by SRM-MS analysis with significant difference in mean expression (p<0.10) between node-negative and node-positive tumour tissue. Proteins displaying significance in more than one SRM transition are marked with an asterisk (*), with minimum p-value and maximum fold-change reported. SRM: Selected reaction monitoring. MS: Mass spectrometry. (DOC) [file pone.0030992.s004.doc]

**Supplemental Table S4**. Differentially expressed proteins identified by SRM analysis.

| **Accession** | **Protein Name** | **Fold-change** | **p** |
| --- | --- | --- | --- |
| **Up-regulated in LN positive group** | | | |
| P51884 | Lumican | 2.5 | 0.001 |
| P02760* | AMBP protein | 2.6 | 0.002 |
| P14625 | Endoplasmin (HSP90B1) | 2.0 | 0.007 |
| P62851* | 40S ribosomal protein S25 | 2.1 | 0.008 |
| O14607 | Ubiquitously transcribed Y chromosome tetratricopeptide repeat protein | 2.4 | 0.030 |
| P69905 | Hemoglobin subunit alpha | 2.3 | 0.036 |
| O00764 | Pyridoxal kinase | 2.4 | 0.036 |
| Q6PGN9 | Proline/serine rich coiled coil protein 1 | 1.9 | 0.045 |
| Q9Y2B0 | MIR interacting saposin like protein | 1.9 | 0.052 |
| P09493 | Tropomyosin alpha 1 chain | 2.0 | 0.054 |
| P08670 | Vimentin | 2.8 | 0.056 |
| P01871 | Ig mu chain C region | 1.9 | 0.057 |
| P02790 | Hemopexin | 3.9 | 0.059 |
| P07585 | Decorin | 1.5 | 0.060 |
| P68032* | Actin alpha cardiac muscle 1 | 1.9 | 0.062 |
| P0C0L5* | Complement C4 B | 2.0 | 0.065 |
| P00450 | Ceruloplasmin | 2.3 | 0.069 |
| P30042 | ES1 protein homolog, mitochondrial | 1.5 | 0.073 |
| P61088 | Ubiquitin conjugating enzyme E2 N | 1.7 | 0.086 |
| P07737* | Profilin 1 | 1.7 | 0.086 |
| P67936 | Tropomyosin alpha 4 chain | 2.1 | 0.093 |
| P08603 | Complement factor H | 1.4 | 0.097 |
| P63313 | Thymosin beta 10 | 9.1 | 0.099 |
| **Down-regulated in LN positive group** | | | |
| P62826 | GTP binding nuclear protein Ran | 1.9 | 0.003 |
| P05204* | Non histone chromosomal protein HMG 179493 | 2.2 | 0.014 |
| P11940 | Polyadenylate binding protein 1 | 3.2 | 0.018 |
| P02765* | Alpha 2 HS glycoprotein | 2.1 | 0.031 |
| P10809* | 60 kDa heat shock protein, mitochondrial | 2.5 | 0.034 |
| Q9UKN7 | Myosin XV | 3.3 | 0.034 |
| Q70CQ2 | Ubiquitin carboxyl terminal hydrolase 34 | 4.1 | 0.035 |
| P63104 | 14-3-3 protein zeta delta | 1.9 | 0.041 |
| P00738* | Haptoglobin | 2.3 | 0.043 |
| P05787 | Keratin type II cytoskeletal 8 | 1.7 | 0.045 |
| P12111 | Collagen alpha 3 VI chain | 2.7 | 0.058 |
| P00352 | Retinal dehydrogenase 1 | 3.4 | 0.063 |
| Q9Y281 | Cofilin 2 | 3.4 | 0.063 |
| P02768 | Serum albumin | 3.8 | 0.065 |
| P06753 | Tropomyosin alpha 3 chain | 1.9 | 0.066 |
| P09382 | Galectin 1 | 2.2 | 0.067 |
| P11413 | Glucose-6-phosphate-1-dehydrogenase | 3.4 | 0.073 |
| P02763 | Alpha-1-acid-glycoprotein-1 | 2.6 | 0.074 |
| O43707 | Alpha actinin 4 | 2.6 | 0.077 |
| P01781 | Ig heavy chain V III region GAL | 2.1 | 0.079 |
| P00338 | L lactate dehydrogenase A chain | 1.7 | 0.081 |
| Q96QF0 | RAB3A interacting protein | 2.3 | 0.085 |
| P22307 | Non specific lipid transfer protein | 1.8 | 0.087 |
| Q86V81 | THO complex subunit 4 | 2.7 | 0.092 |
| P08519 | Apolipoprotein(a) | 1.6 | 0.095 |
| Q06830 | Peroxiredoxin 1 | 1.7 | 0.095 |

Proteins identified by SRM-MS analysis with significant difference in mean expression (p < 0.10) between node-negative and node-positive tumour tissue. Proteins displaying significance in more than one SRM transition are marked with an asterisk (*), with minimum p-value and maximum fold-change reported

SRM: Selected reaction monitoring

MS: Mass spectrometry
