# Supplementary figures and images for: Proteomic Analyses Reveal High Expression of Decorin and Endoplasmin (HSP90B1) Are Associated with Breast Cancer Metastasis and Decreased Survival
Source: PLoS One. 2012 Feb 20;7(2):e30992. doi: 10.1371/journal.pone.0030992 (PMC3282708; doi:10.1371/journal.pone.0030992)

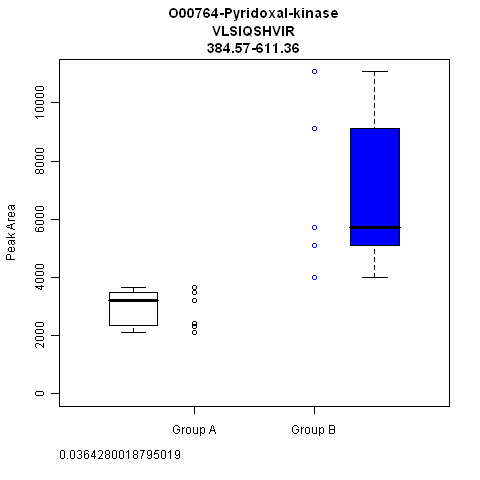

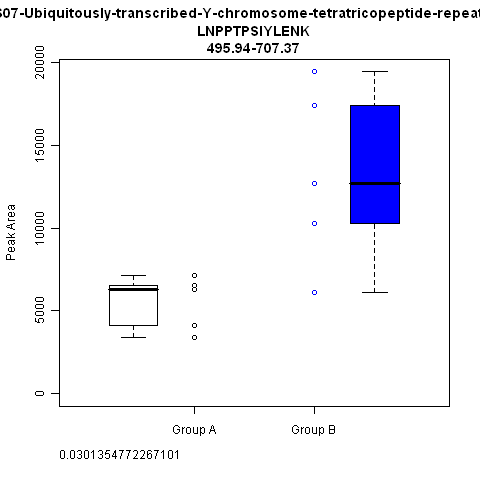


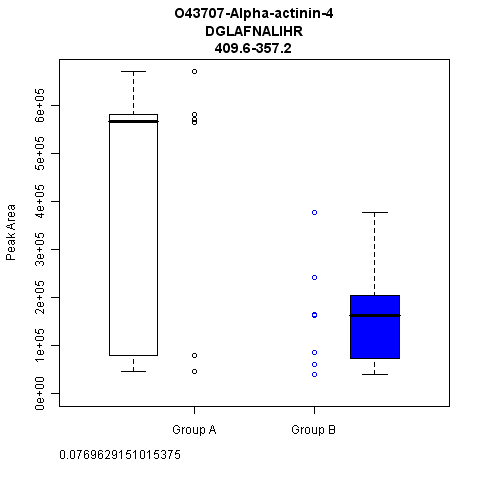

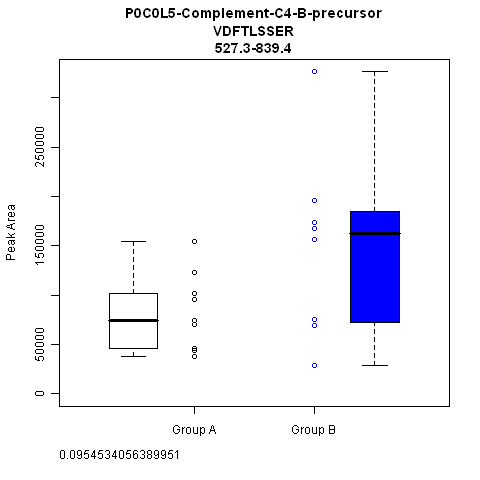


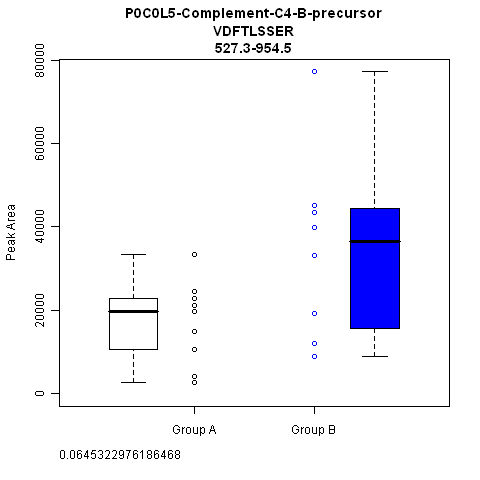

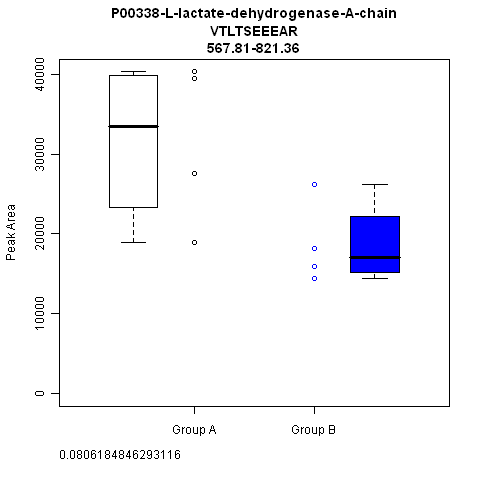


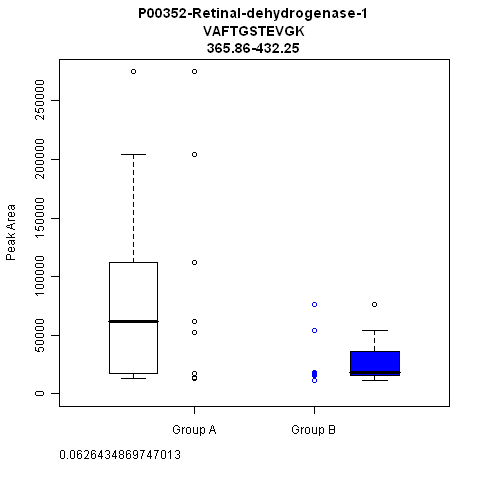

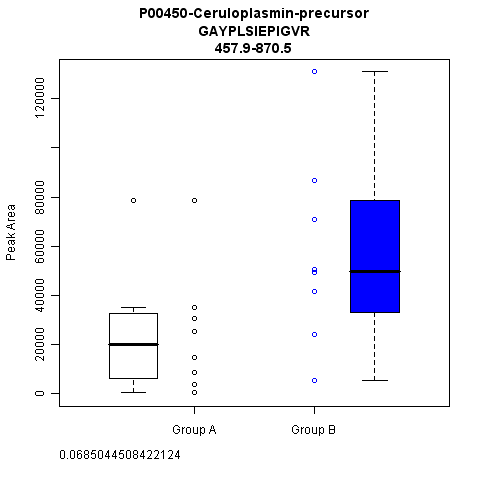


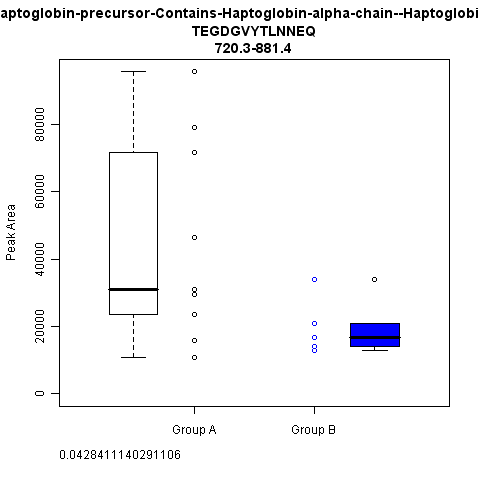

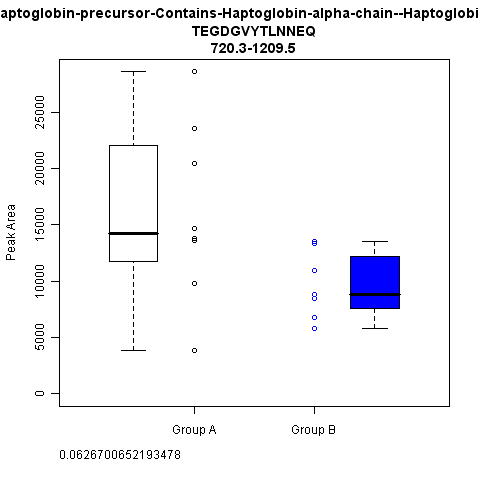


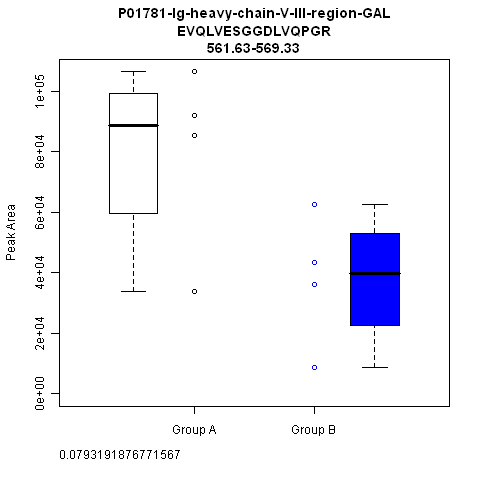

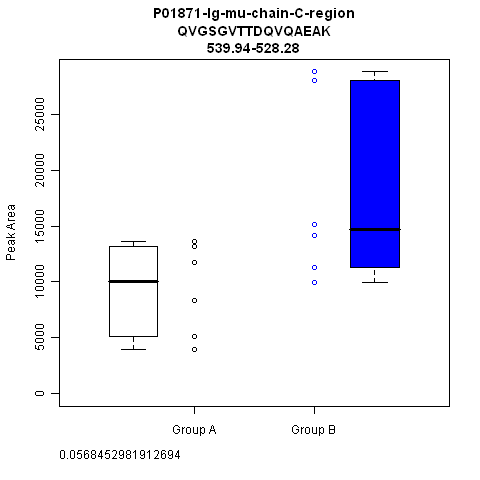


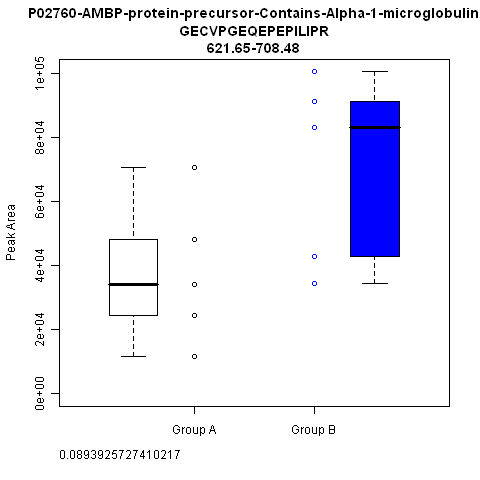

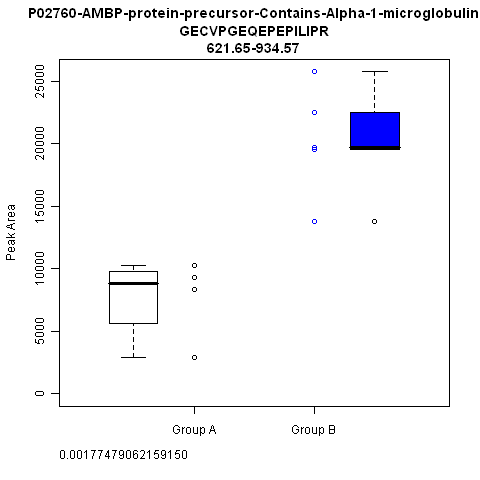


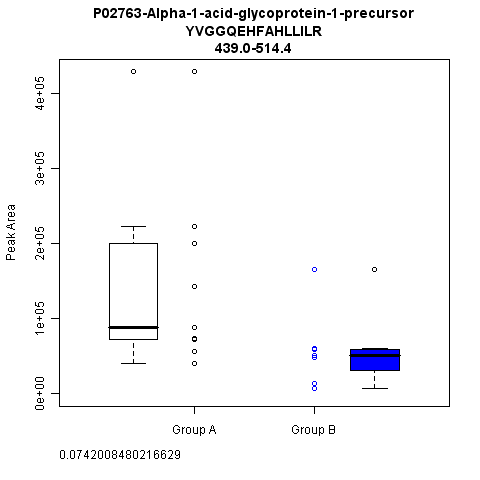

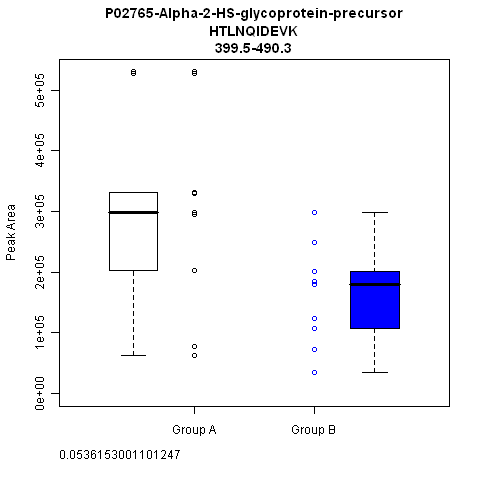


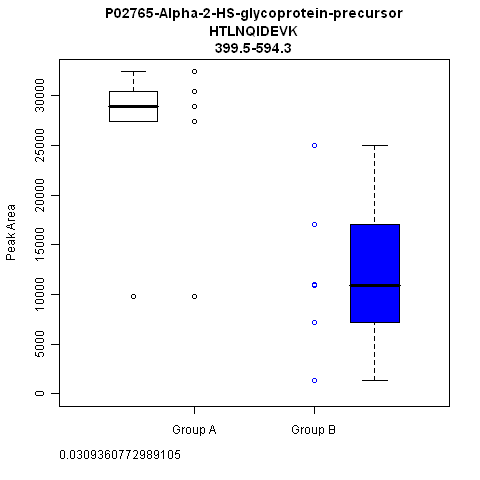

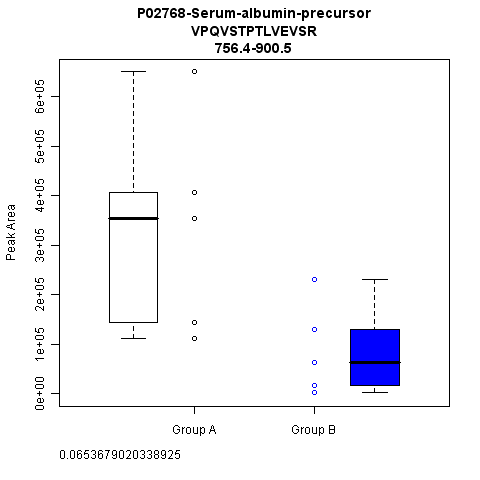


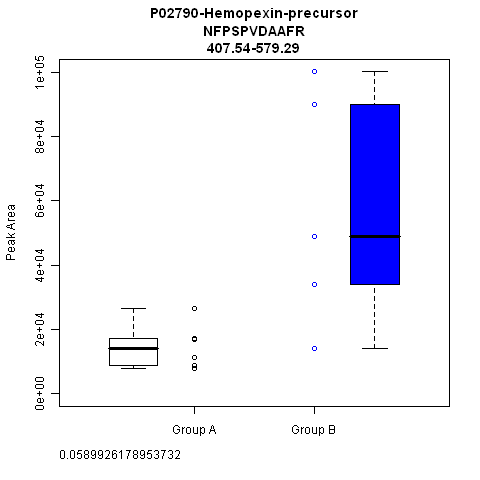

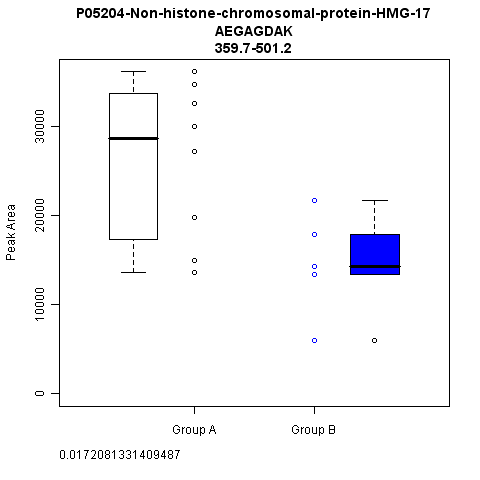


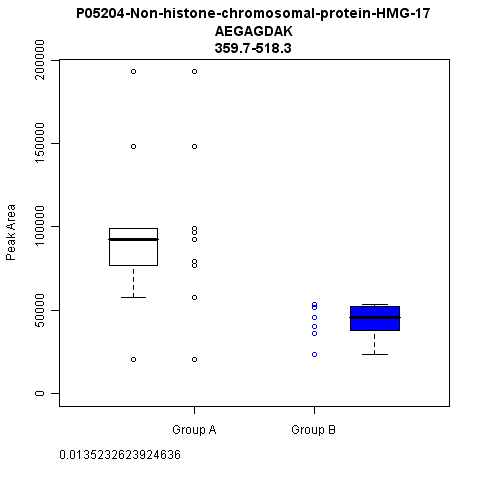

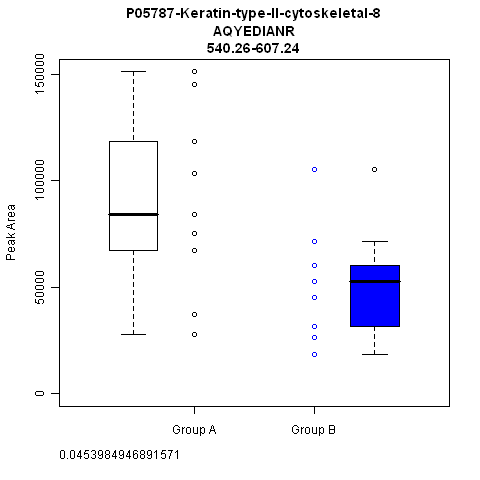


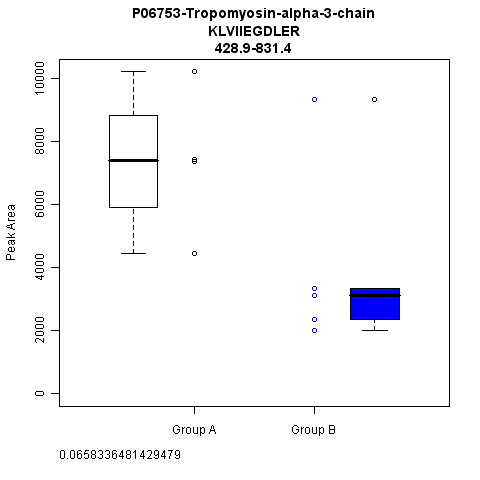

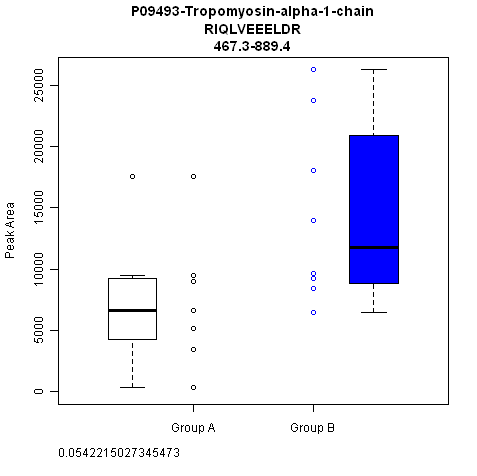


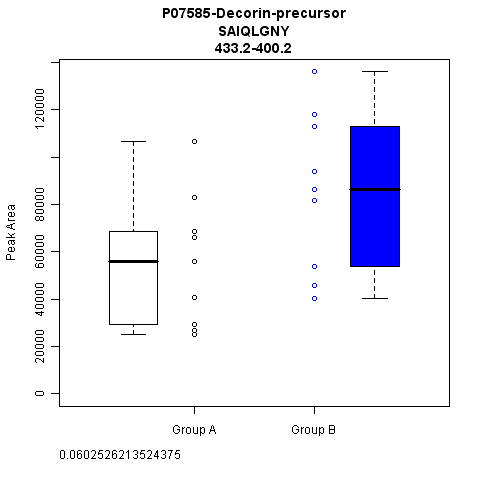

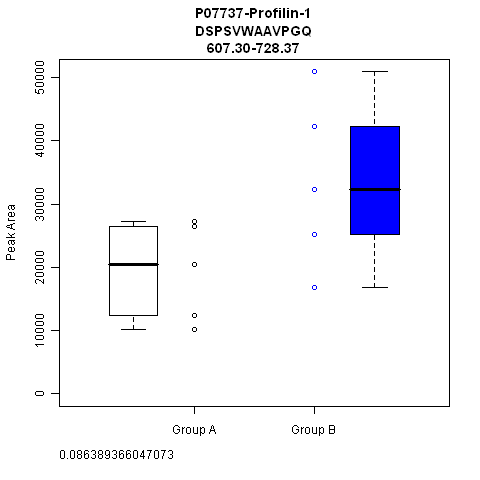


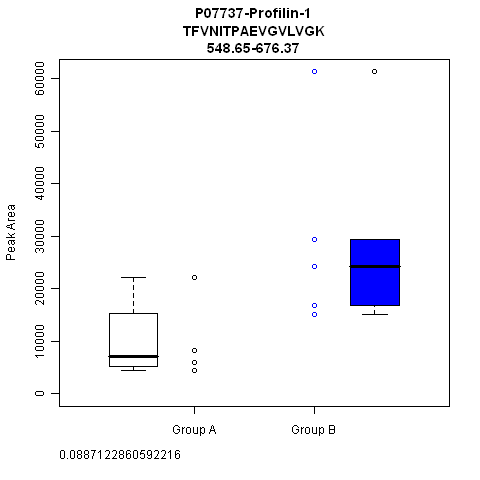

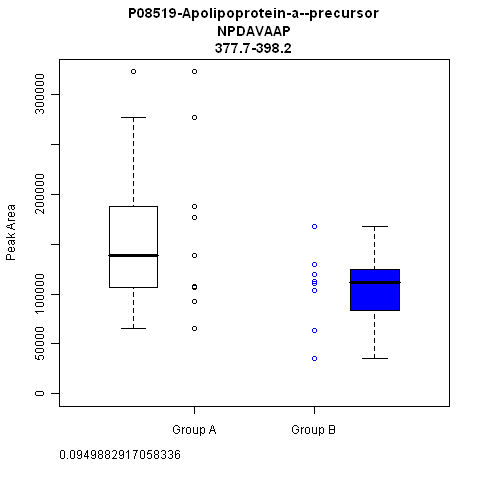

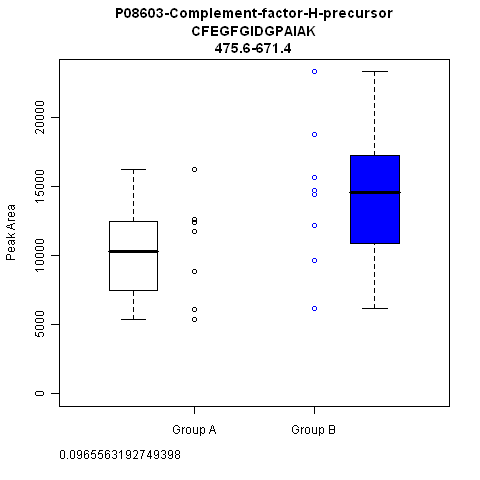

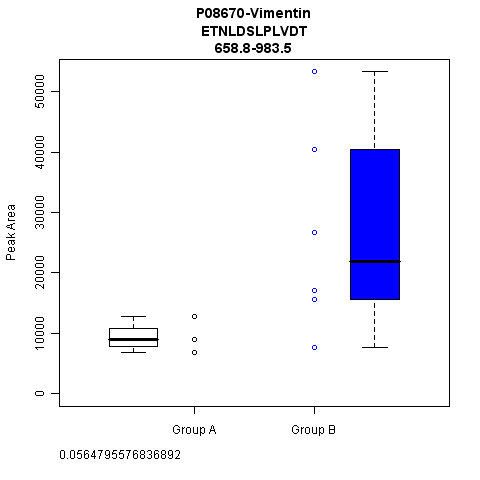

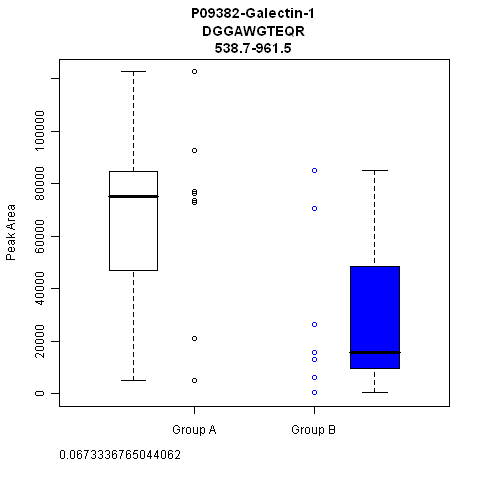

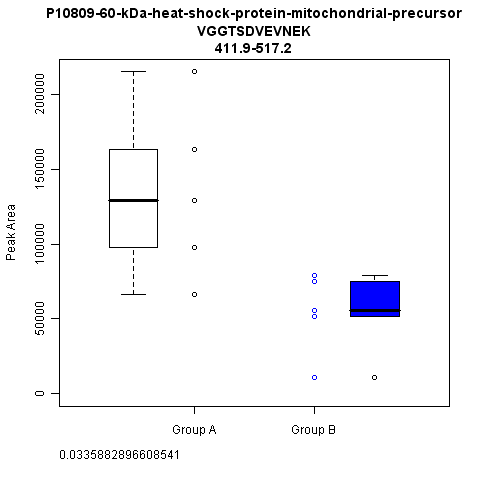

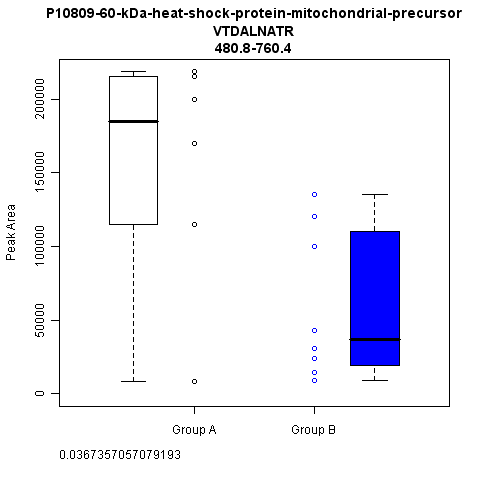

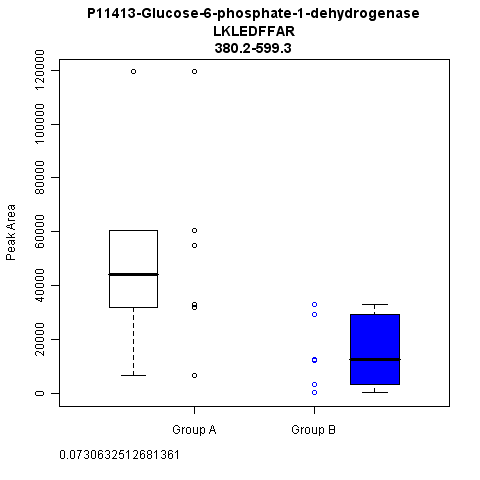

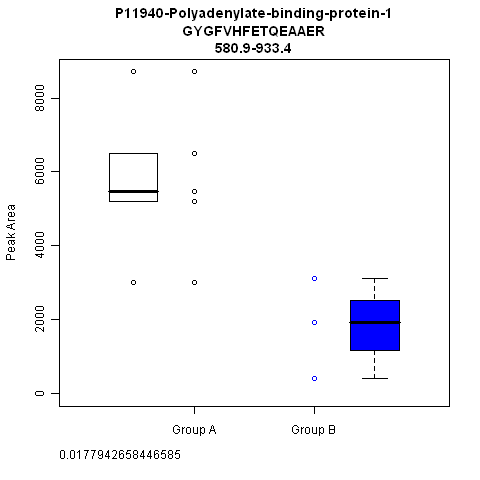

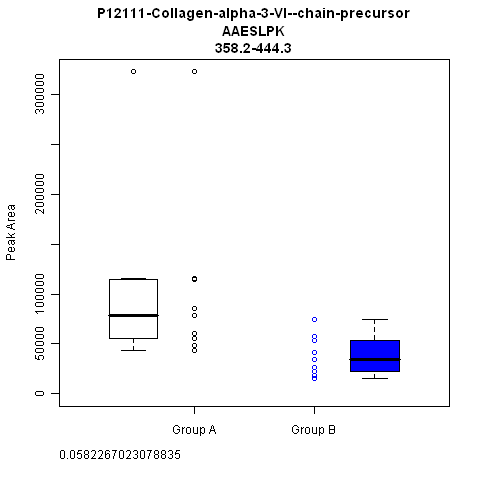


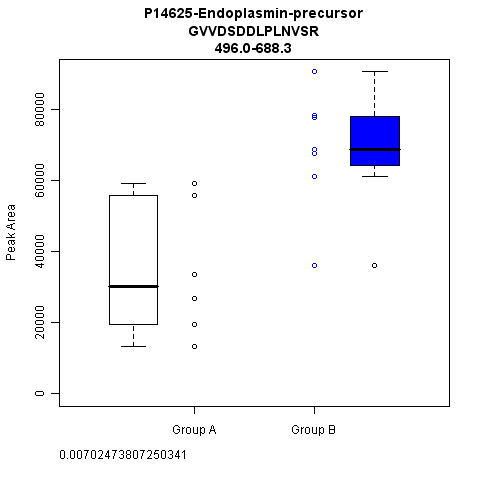

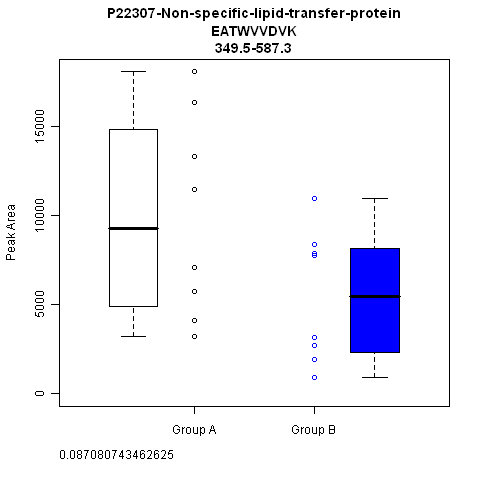


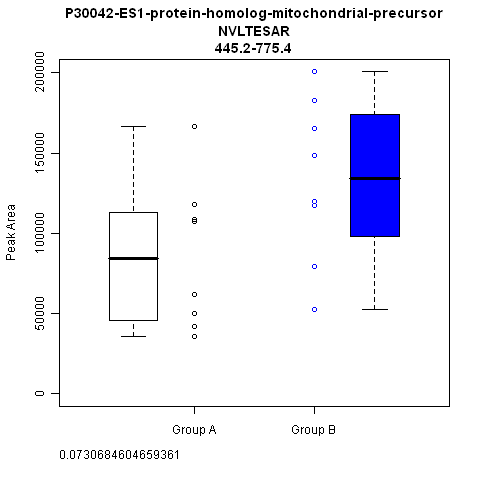

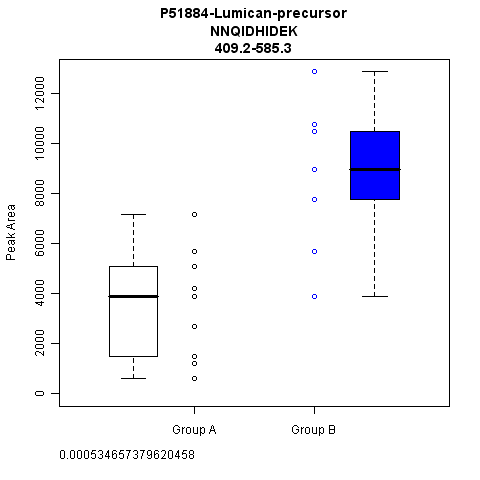


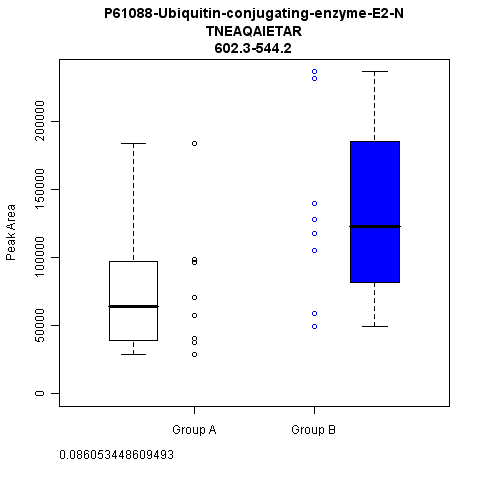

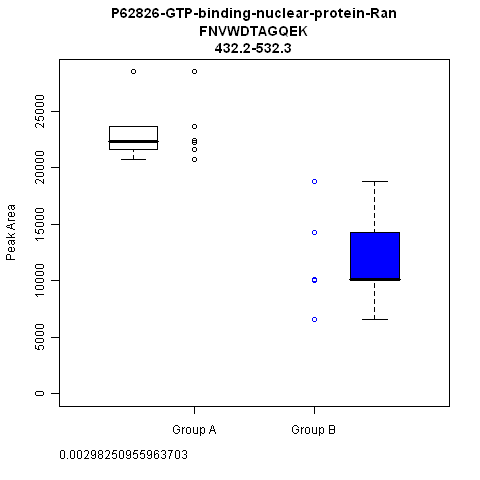


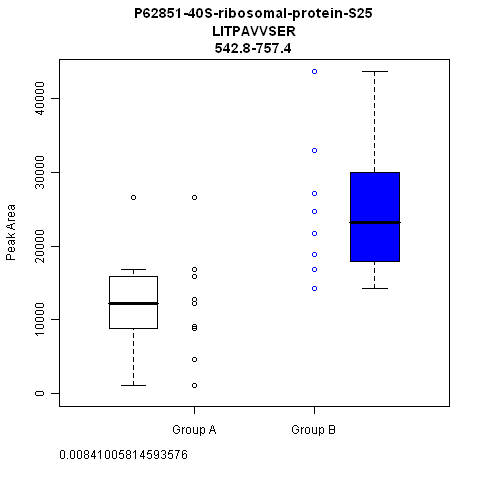

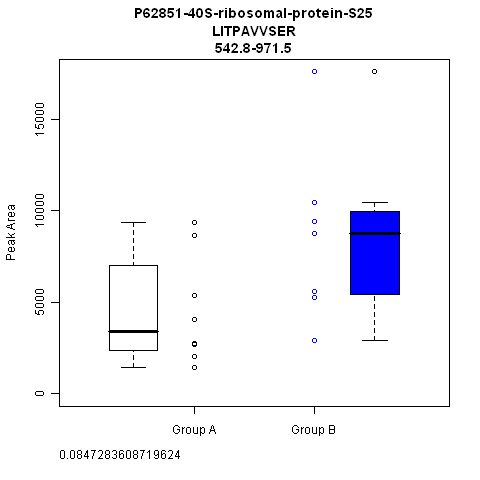


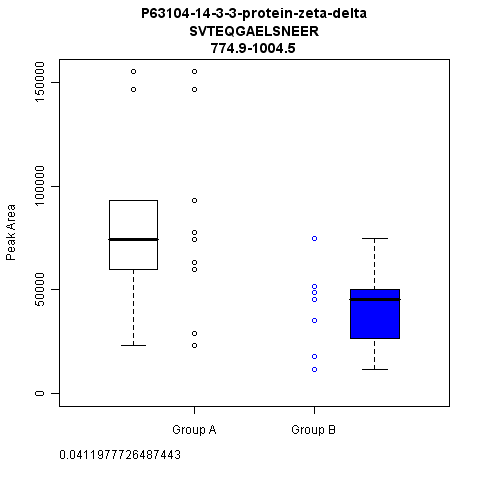

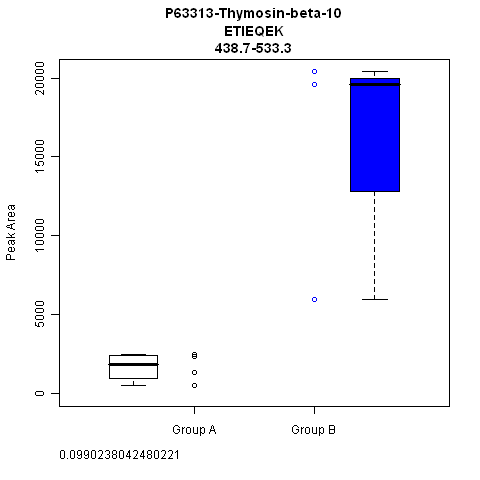


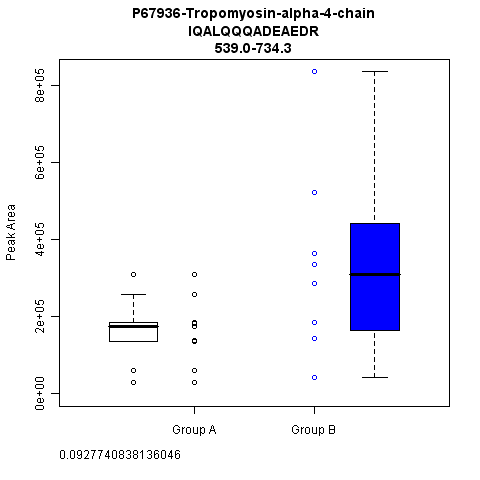

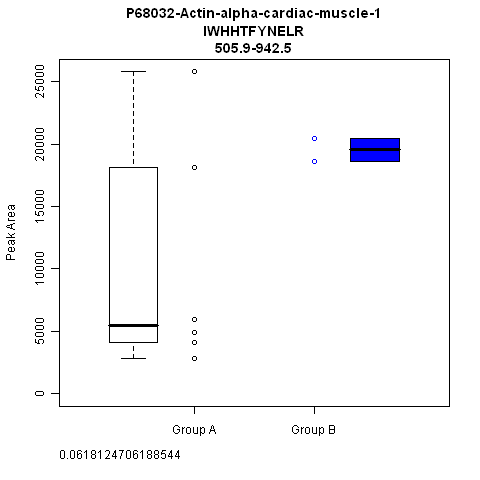


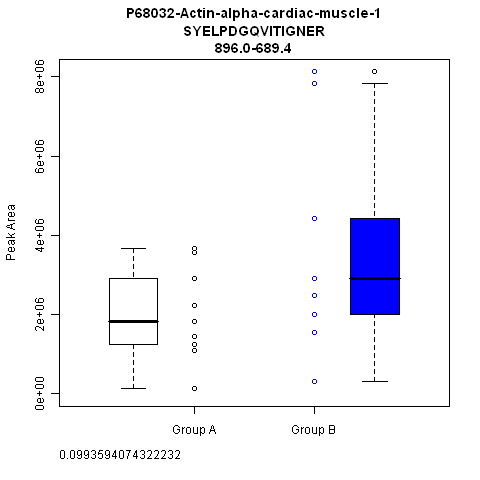

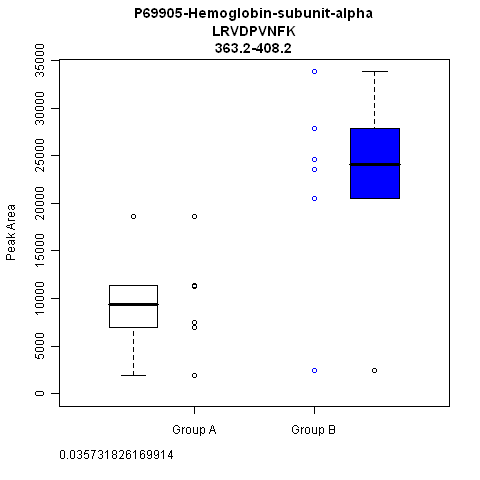


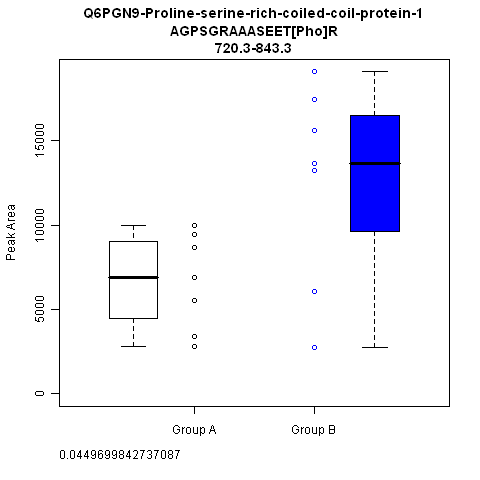

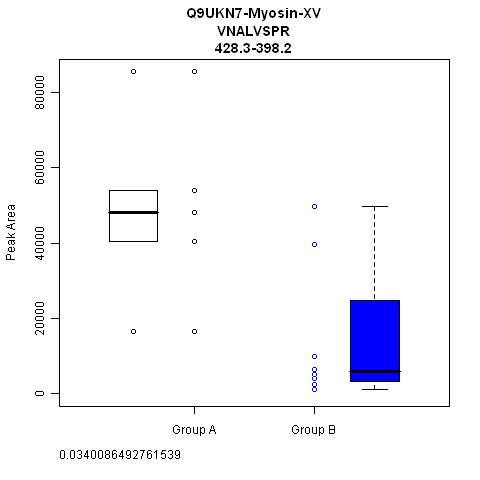


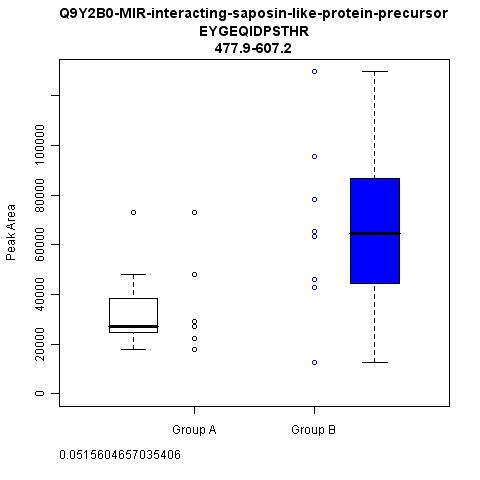

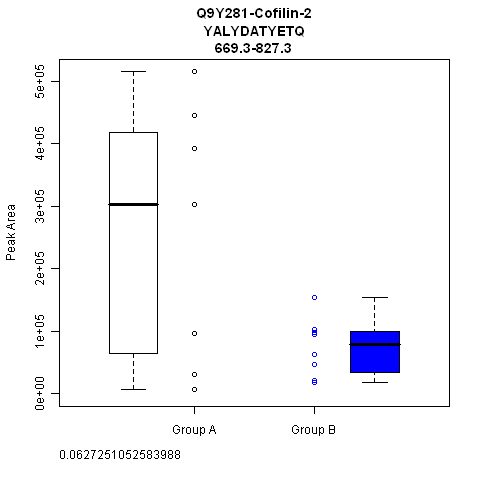


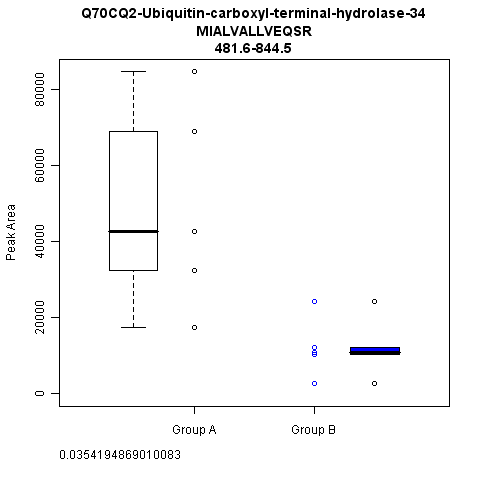

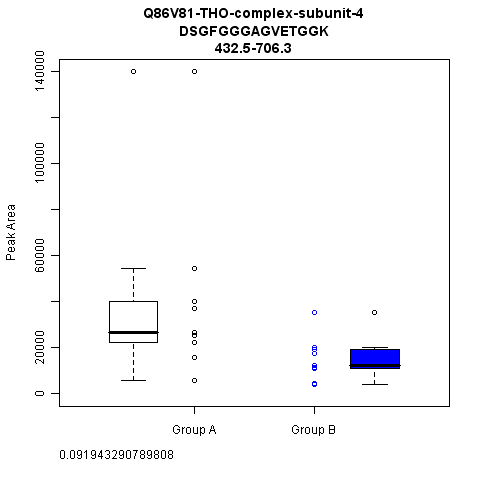


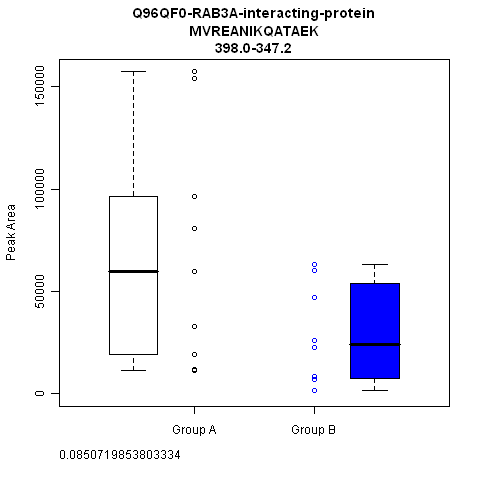

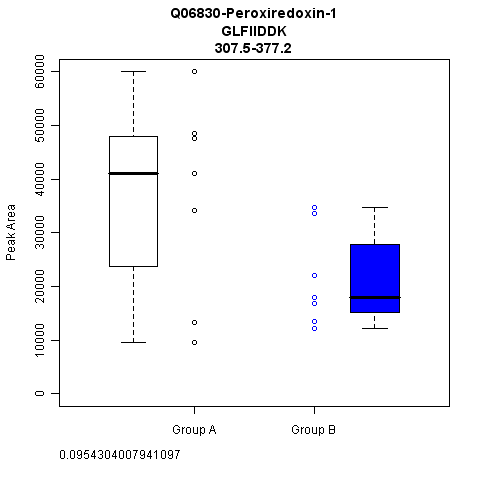

Supplement: Data S1 — Box plots summarizing peak values of proteins identified by SRM-MS analysis. SRM-MS analysis identified 49 proteins with significant difference in mean expression (p<0.10) between node-negative and node-positive tumour tissue. SRM: Selected reaction monitoring. MS: Mass spectrometry. (DOC) [file pone.0030992.s005.doc]
